# Exported Proteins Required for Virulence and Rigidity of *Plasmodium falciparum*-Infected Human Erythrocytes

**DOI:** 10.1016/j.cell.2008.04.051

**Published:** 2008-07-11

**Authors:** Alexander G. Maier, Melanie Rug, Matthew T. O'Neill, Monica Brown, Srabasti Chakravorty, Tadge Szestak, Joanne Chesson, Yang Wu, Katie Hughes, Ross L. Coppel, Chris Newbold, James G. Beeson, Alister Craig, Brendan S. Crabb, Alan F. Cowman

**Affiliations:** 1 The Walter and Eliza Hall Institute of Medical Research, Melbourne 3050, Australia; 2 Liverpool School of Tropical Medicine, Liverpool L3 5QA, UK; 3 Monash University, Department of Microbiology, Clayton 3800, Australia; 4 University of Oxford, Weatherall Institute of Molecular Medicine, John Radcliffe Hospital, Oxford OX3 9DS, UK

**Keywords:** HUMDISEASE, CELLBIO

## Abstract

A major part of virulence for *Plasmodium falciparum* malaria infection, the most lethal parasitic disease of humans, results from increased rigidity and adhesiveness of infected host red cells. These changes are caused by parasite proteins exported to the erythrocyte using novel trafficking machinery assembled in the host cell. To understand these unique modifications, we used a large-scale gene knockout strategy combined with functional screens to identify proteins exported into parasite-infected erythrocytes and involved in remodeling these cells. Eight genes were identified encoding proteins required for export of the parasite adhesin PfEMP1 and assembly of knobs that function as physical platforms to anchor the adhesin. Additionally, we show that multiple proteins play a role in generating increased rigidity of infected erythrocytes. Collectively these proteins function as a pathogen secretion system, similar to bacteria and may provide targets for antivirulence based therapies to a disease responsible for millions of deaths annually.

## Introduction

*Plasmodium falciparum* causes the most severe form of malaria in humans with 1 to 3 million deaths annually. Once in the blood, multiplication of the parasite inside erythrocytes is responsible for associated morbidity and mortality. Profound structural and morphological changes occur in erythrocytes after parasite invasion, dramatically altering their physical properties and impairing circulation in vivo (Cooke et al., 2004). In contrast to normal erythrocytes, parasitised cells are rigid and adhere to host endothelium as well as other cell types (Barnwell, 1989). The increased rigidity and adhesiveness of *P. falciparum*-infected erythrocytes result in augmented haemodynamic resistance in the microvasculature (Raventos et al., 1985) and play an important role in the pathogenesis of malaria.

Adherence of infected red cells to vascular endothelium is mediated by *P. falciparum* erythrocyte membrane protein (PfEMP1) (Leech et al., 1984), an antigenically diverse protein family trafficked to the infected red cell surface (Baruch et al., 1995; Smith et al., 1995; Su et al., 1995). This in turn is anchored at the red cell membrane skeleton by knobs, macromolecular complexes consisting of knob associated histidine-rich protein (KAHRP) (Crabb et al., 1997). In the absence of knobs, PfEMP1 cannot form adhesive interactions of sufficient strength to withstand disruption by forces of blood flow (Crabb et al., 1997). KAHRP binding with the membrane skeleton leads to an increased rigidity, blockage of blood vessels and resistance to flow (Pei et al., 2005). The parasite proteins involved are transported through host cells without trafficking machinery and inserted into a highly organized membrane skeleton structure. The formation of a de novo transport system and trafficking of parasite proteins to diverse locations in the host cell is unique in cell biology (Marti et al., 2005).

Parasite proteins such as PfEMP1 and KAHRP have to traverse several membranes to reach their destination (Marti et al., 2005). A pentameric sequence has been identified required for translocation of proteins across the parasitophorous vacuole membrane termed the *P. falciparum* Export Element (PEXEL) (Marti et al., 2004) or Vacuolar Targeting Signal (VTS) (Hiller et al., 2004). Indeed, a similar sequence has been identified in the parasitic fungi *Phytophtora infestans* that is required for export of proteins into infected plant cells (Whisson et al., 2007). Searching of the *P. falciparum* genome sequence has revealed 8% of *P. falciparum* genes contain this sequence (Hiller et al., 2004; Marti et al., 2004; Sargeant et al., 2006). Many of these are likely to encode proteins that play an important role in remodelling infected erythrocytes (Marti et al., 2005).

Translocation across the parasitophorous vacuole membrane via a PEXEL motif is functionally conserved across all *Plasmodium* species. However the ‘exportome’ for *P. falciparum* is 5-10 times larger than that of other malaria parasites partly because of radiation and expansion of gene families including those containing DnaJ domains (Walsh et al., 2004) and other novel domains called PHIST (*Plasmodium* helical interspersed subtelomeric family) (Sargeant et al., 2006). One explanation for increased number of proteins exported to the host erythrocyte in *P. falciparum* is they are necessary for export of *P.falciparum* specific PfEMP1 to the parasite-infected erythrocyte surface (Marti et al., 2005). Once across the parasitophorous vacuole, many exported proteins interact with novel structures in the red cell cytoplasm called Maurer's clefts, structures that serve as a sorting point from which *P. falciparum* proteins are deposited underneath or into the erythrocyte membrane (Wickham et al., 2001). At least one of the proteins resident in clefts, the skeleton binding protein 1 (SBP1) has been shown to be required for transport of PfEMP1 to the red cell membrane (Cooke et al., 2006; Maier et al., 2007).

To identify proteins involved in this process we used functional screens by constructing loss-of-function mutants of genes encoding proteins predicted to be exported. We were particularly interested in finding proteins required for trafficking PfEMP1 to the infected erythrocyte surface, correct assembly of knobs and those involved in rigidification of infected red cells, all processes associated with virulence in malaria infection. The scale of these studies is an order of magnitude greater than previously attempted in the field of malaria. This allowed us to identify previously unknown proteins exported to the *P. falciparum*-infected erythrocyte, responsible for establishment of the parasite in its intracellular environment and providing essential functions for assembly and localization of virulence determinants.

## Results

### Generation of Loss-of-Function Parasites Lacking Expression of Exported Proteins

**Figure 1:**
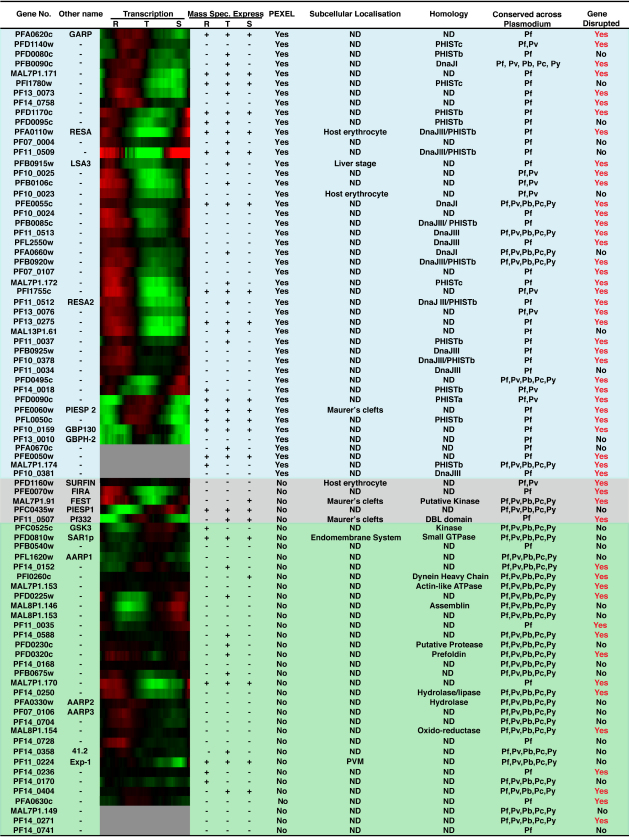
Expression and Homology of Genes Selected for Gene Knockout Screen in *P. falciparum* The *P. falciparum* genome was scanned to generate a list that included known exported proteins, as well as those with a PEXEL motif (Hiller et al., 2004; Marti et al., 2004; Sargeant et al., 2006). Using these criteria we compiled a list of 83 candidate genes of which 46 had PEXEL motifs (shaded blue). Those not containing a PEXEL but known to be exported are shaded gray. Genes shaded green are not known to be exported or are present on the parasitophorous vacuole membrane (PVM) e.g. Exp-1 (PF11_0224). Shown in the first column is the gene number which can be found at http://www.plasmodb.org. The second column refers to a known name of the corresponding protein. The third column shows a transcription profile where red is an increased period of transcription and green a decreased period or no transcription. Gene transcription was obtained from the microarray data available on http://malaria.ucsf.edu/ (Bozdech et al., 2003). In the fourth column proteomic data is shown and + refers to peptides detected in ring (R), trophozoite (T) or schizont (S) asexual life cycle stages. Proteomic data is available on www.plasmodb.org. The fifth column refers to the presence (Yes) or absence (No) of a Plasmodium export element or PEXEL (Hiller et al., 2004; Marti et al., 2004; Sargeant et al., 2006). The sixth column refers to the subcellular localization of the protein in the *P. falciparum*-infected erythrocyte if known and if not known shown as ND. The seventh column shows the homology detected and in some cases putative function of the protein and where none was detected is shown as ND. The eighth column shows the conservation of genes within different Plasmodia spp.: Pf, *P. falciparum*; Pv, *P. vivax*; Pb, *P. berghei*; Pc, *P. chabaudi*; Py, *P. yoelii*. The ninth column refers to whether the gene can be genetically disrupted (Yes) or not (No).

We scanned the *P. falciparum* genome to generate a list that included known exported proteins, as well as those with a PEXEL motif (Hiller et al., 2004; Marti et al., 2004; Sargeant et al., 2006). Using these criteria we compiled a list of 83 candidate genes of which 46 had PEXEL motifs (Figure 1, shaded blue). Five genes were chosen that do not have a PEXEL but are exported including SURFIN (Winter et al., 2005), FIRA (Stahl et al., 1987), FEST (Kun et al., 1997), PIESP1 (Florens et al., 2004) and Pf332 (Mattei and Scherf, 1992) (Figure 1, shaded gray). Together, these 51 exported proteins constitute a representative subset of the exportome manageable in terms of a *P. falciparum* gene knockout screen. In addition, we included 32 genes encoding proteins with a signal sequence and gene transcription in blood-stages to provide a comparison with respect to essentiality (Figure 1, shaded green). The original list was made before identification of the PEXEL motif. The latter gene set were identified as potentially exported as they fitted bioinformatic criteria including a signal sequence and transcription in early rings. Subsequently, upon identification of the PEXEL the list was refined; however, we retained the 32 genes classed as not exported. Most genes within the exported set were transcribed either in ring stages soon after invasion and/or in schizont stages when the invasive merozoite is formed (Figure 1). This is consistent with these proteins playing a role in repairing or remodelling the host erythrocyte after invasion of the merozoite.

**Figure 2:**
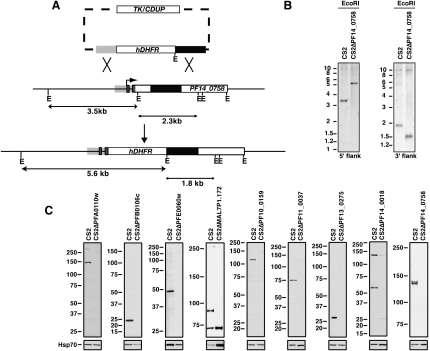
Genetic Disruption of Genes Encoding Exported Proteins (A) Strategy to delete genes as exemplified for PF14_0758. The vectors used were pHHT-TK (TK) or pCC1 (CDUP). The restriction enzyme used for PF14_0758 was EcoRI (E). The flanks for recombination are shaded gray or black. These flanks were probes for Southern blots and the size of DNA fragments based on 3D7 sequence are indicated in kilobases (kb). (B) Example of a Southern blot to verify disruption for PF14_0758 (all disrupted genes are shown in Fig. S1). (C) Western blots to confirm absence of protein expression in parasites lines in which the genes PFA0110w, PFB0106c, PFE0060w, MAL7P1.172, PF10_0159, PF11_0037, PF13_0275, PF14_0018 and PF14_0758 had been disrupted. CS2 is shown in each panel and equal loading demonstrated with Hsp70 antibodies (bottom panel).

To disrupt the function of these genes in *P. falciparum*, we constructed plasmids that integrate into targeted genes by double crossover homologous recombination using plasmid pHHT-TK (Duraisingh et al., 2002) (Figure 2A). During this work we developed improved plasmids (pCC1, see supplementary methods) for negative selection using the *Saccharomyces cerevisiae* cytosine deaminase/uracil phosphoribosyl transferase (CDUP) gene (Figure 2A) (Maier et al., 2006). The plasmids were transfected into CS2, a strain of *P. falciparum* conferring adhesion of infected erythrocytes to chondroitin sulfate A (CSA) via a PfEMP1 encoded by *var2csa* (Salanti et al., 2004). This parasite line was chosen because expression of PfEMP1 encoded by *var2csa* is very stable over time. As most PfEMP1 genes undergo rapid transcriptional switches to other family members as a means of immune evasion these switching events could confound our subsequent analysis, the choice of *var2csa* minimizes this problem.

In *P. falciparum* the transfected plasmids are maintained as episomal circles and integration by double crossover homologous recombination occurs at low frequency (Maier et al., 2006). Growth on WR99210 (positive selection) and 5′-fluorocytosine (negative selection) favors the survival of transfected parasites with homologous integration into the target gene and loss of episomal plasmids (Maier et al., 2006). Gene disruption was analyzed by Southern blots that indicated the plasmid had integrated by double-crossover homologous recombination into 53 of 83 genes (Figures 2B and S1). To show that gene disruption results in loss of protein expression we generated antibodies to a subset and analyzed them by Western blots to demonstrate loss of protein expression (Figure 2C). Although transfection of the plasmids was successful for the other 30 genes, it was not possible to derive parasites in which the constructs had integrated. While the inability to select for double crossover homologous recombination for some genes is not definitive proof that they are essential under laboratory conditions it is consistent with the proposition that they serve an important function in growth of the parasite in the host erythrocyte.

### “Essentiality” of Exported Proteins in *P. falciparum*

**Figure 3:**
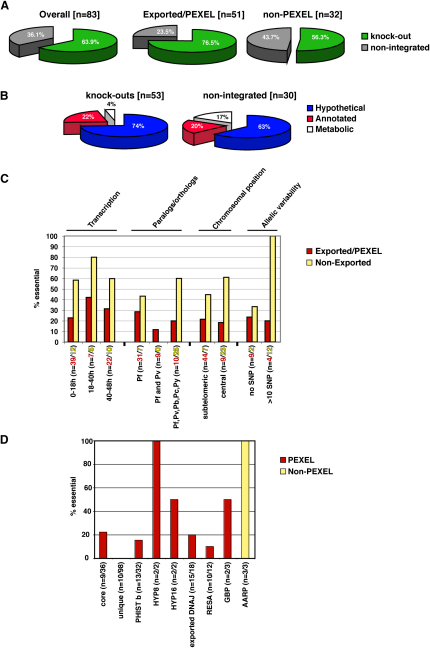
Essentiality of Genes in *P. falciparum* (A) Comparison of essentiality for genes judged by ability to genetically disrupt them. Comparison is shown for all genes (Overall), the exported and PEXEL containing proteins (Exported/PEXEL) and those not exported (non-PEXEL). Unsuccessful disruptions are in gray while successful ones are in green. (B) Comparison of non-essential proteins to those not disrupted. The proteins are divided into hypothetical (blue), annotated (red) and those assigned to a metabolic pathway (white) (PlasmoDB, www.plasmodb.org). (C) Essentiality of different gene groups. The essentiality of the genes was compared with respect to transcription profile, homologies, chromosomal position and allelic variability. The bars show essentiality (as determined by the percentage of unsuccessful gene knock-outs for each group) of exported (red) and non-exported gene products (yellow). n = number of attempted gene disruption for exported (red) and non-exported proteins (yellow) in each group. (D) The essentiality of gene families as shown by the ability to generate a genetic disruption. n = number of attempted gene disruption/total members in gene family. PEXEL-containing families are shown in red and non-PEXEL families are depicted in yellow.

We propagated *P. falciparum* in human erythrocytes in vitro and under these circumstances, genes that may be essential for survival in vivo (e.g., in the presence of the immune system), such as those involved in the transport of PfEMP1 and its display on the parasite-infected erythrocyte surface, may not be required. Therefore we expected fewer exported proteins to be essential for in vitro growth when compared to non-exported blood-stage proteins, many of which presumably function to maintain normal erythrocytic growth. Overall, 53 of the *P. falciparum* genes tested could be disrupted and classified as non-essential for erythrocytic growth (64% of those tested) (Figure 3A). Consistent with our hypothesis, fewer exported proteins were likely to be essential (23.5%) than those not exported from the parasite (43.7%). Genes encoding proteins annotated as having a probable metabolic role were over-represented among “essential” genes whereas other annotated classes or those with no obvious functional homologs (hypothetical proteins) were present in similar proportions in the gene knockout and essential groups (Figure 3B).

Interestingly, for genes *PFD0095c, MAL7P1.149* and *MAL8P1.153* we were able to disrupt the endogenous loci but this was accompanied by a duplication event maintaining expression of the gene (Figure S2). We concluded these genes are essential for in vitro growth. No matter which sub-classification was used to group different genes, a higher proportion of non-exported proteins were considered essential (Figure 3C). Among the genes encoding exported proteins, both disruptable and non-disruptable examples were found within the PHIST family and those containing DnaJ domains. The latter suggests that some co-chaperone functions may be essential, while others may not (Walsh et al., 2004) (Figure 2D).

### Identification of Genes Required for PfEMP1 Surface Expression

**Figure 4:**
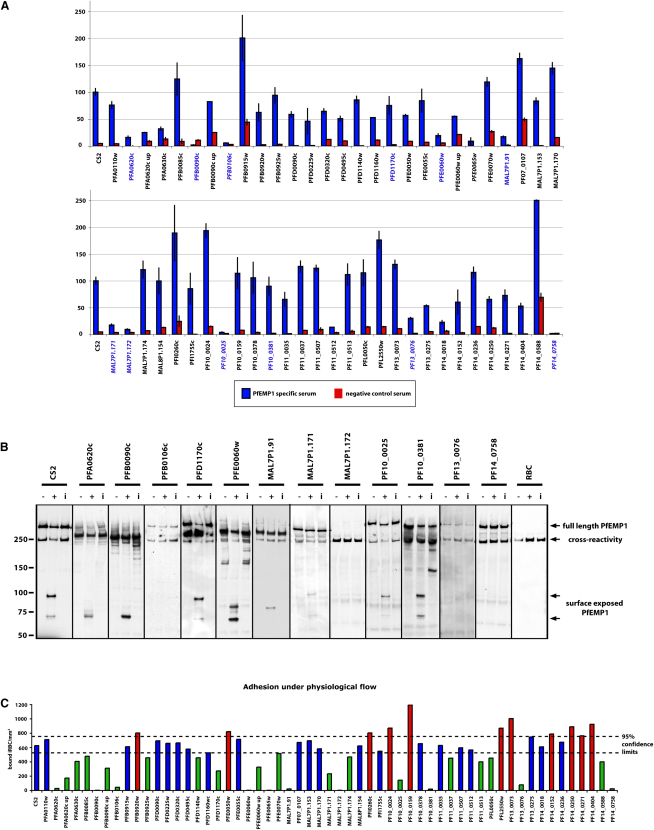
Identification of Proteins Required for Display and Function of PfEMP1 on the Surface of *P. falciparum*-Infected Erythrocytes (A) Screening of mutant parasite strains to identify those with altered reactivity to anti-var2csa antibodies by FACS. Mutant parasite lines with specific gene disruptions were tested for reactivity with serum antibodies from malaria-exposed multigravid women (blue bars) and non-exposed controls (red bars). Reactivity was expressed as relative to the parental line CS2, which was set at 100%. Gene names in blue signify candidates for a trypsin cleavage assay (Figure 3B) and gene names in italics indicate a subsequently identified influence on PfEMP1 transport (Figure 7). Error bars indicate % range. (B) Trypsin treatment of *P. falciparum*-infected erythrocytes to determine presence of PfEMP1 on the host erythrocyte. The full-length PfEMP1 and cytoplasmic tail were detected using antibodies to the cytoplasmic acidic terminal segment (ATS). Full-length PfEMP1 was a > 300 kDa band. Surface pool of PfEMP1 was detected by a trypsin-resistant band between 70 and 90 kDa. The lanes in each panel show Triton-X insoluble/SDS soluble extractions of parasite-infected erythrocytes: first lane, untreated (−); second lane, trypsin-treated (+); third lane, trypsin plus soybean trypsin inhibitor (i). The parasite lines shown are those that when screened by antibodies against var2csa PfEMP1 were less than 30% reactive compared to CS2 or knob-deficient (panel A). The red cell control is shown in the last panel. The anti-ATS antibody cross-reacts with spectrin (Maier et al., 2007; Rug et al., 2006). Lack of a band between 70 and 90 kDa in the trypsin-treated lanes shows absence of PfEMP1 on the erythrocyte surface. Full-length PfEMP1 is observed because there is a large pool of internal protein resistant to trypsin (Waterkeyn et al., 2000). (C) Adherence of mutant *P. falciparum*-infected erythrocytes to CSA under flow conditions. Each of the *P. falciparum* mutant strains was tested for binding to CSA under flow conditions and parasitised cells counted as bound infected red blood cells/mm^2^. 95% confidence intervals for CS2 wild-type binding is presented (dashed lines).

To identify genes required for trafficking, display and function of PfEMP1 on the surface of *P. falciparum*-infected erythrocytes we screened mutant lines for recognition of surface antigens by antibodies from malaria-exposed individuals (Beeson et al., 2006). The CS2 parasite line used here expresses the *var2csa* gene (PFL0030c) (Duffy et al., 2006), which encodes a PfEMP1 responsible for adhesion to CSA (Salanti et al., 2004). In vivo, parasites expressing this *var* gene tend to be found in primigravid women and very rarely in males or mutligravid women who have developed antibodies specific for the *var2csa* PfEMP1. We can thus use sera from multigravid women to detect surface expressed PfEMP1 on CS2-infected erythrocytes (Beeson et al., 2006). An initial screen with such sera showed that 10 of the 53 parasite-infected erythrocyte lines had a decrease in reactivity of ≥ 70% compared to parental CS2-infected red cells (Figure 4A) and we chose these as a cut off for further analyses.

To confirm the reduced level of PfEMP1 on the surface of these cells we used an assay in which surface exposed protein is cleaved by trypsin and the conserved C terminus of the protein detected by western blot with antibodies against the acidic terminal segment (ATS) (Waterkeyn et al., 2000). This differentiates surface exposed PfEMP1 from the intracellular pool. Surface exposed PfEMP1 in parental line CS2 results in cleavage products of 90 and 70 kDa (Figure 4B) whereas the internal pool of PfEMP1 in these cells migrates at approximately 300 kDa. This antibody also shows crossreaction with host spectrin (Rug et al., 2006). Four of the mutant cell lines, in which the genes *PFB0106c, MAL7P1.172, PF13_0076* and *PF14_0758* had been disrupted, showed none or very low levels of surface expressed PfEMP1 as evidenced by the absence of cleaved fragments of any size. The CS2ΔMAL7P1.172 cells also showed greatly reduced levels of total PfEMP1. The parasite lines CS2ΔMAL7P1.171 and CS2ΔPF10_0025 showed consistently reduced surface expression of PfEMP1, in multiple independent experiments, in comparison with parental wild-type cells. These results suggest that proteins encoded by *PFB0106c, MAL7P1.172, PF13_0076, PF14_0758, MAL7P1.171* and *PF10_0025* play a role in trafficking and display of the virulence protein PfEMP1 on the host erythrocyte (Figure 7).

Although the expression of the *var2csa* gene is very stable in comparison to other members of the *var* gene family, switching to other *var* genes does occur at low frequency and these other *var* gene products would not react with the sera that we used. In order to eliminate such false positives, we screened all knockout parasites for their ability to bind to CSA, which is a unique feature of the *var2sa* gene product. Four parasite lines CS2ΔPFA0620c, CS2ΔPFB0090c, CS2ΔPFE0060w and CS2ΔMAL7P1.91 showed decreased levels of adherence to CSA and a trypsin-cleaved C terminus of PfEMP1 of altered size suggesting a switch to an alternative PfEMP1-encoding gene. These lines were subjected to selection for CSA binding to recover parasites in which *var2csa* was the dominant *var* gene expressed. Following selection, an increased reactivity with human serum from multigravid females compared to unselected lines was observed (Figure 4A PFA0620c up, PFB0090c up, PFE0060w up). Additionally, the size of the trypsin-cleaved PfEMP1 was now the same as the parental CS2 line (Fig. S3 compared to before CSA selection Figure 4C). We conclude that these lines are false positives due to antigenic switching.

### Identification of Mutant *P. falciparum* Lines that Show Altered Adherence Properties

To confirm the lines in which the genes *PFB0106c, MAL7P1.172, PF14_0758, MAL7P1.171, PF10_0025,* and *PF13_0076* were disrupted had altered adherence properties, and to identify others in which adherence had been affected, we used flow based cytoadherence assays with CSA. The parasite lines CS2ΔPFB0106c, CS2ΔMAL7P1.172, CS2ΔPF14_0758 show no adherence to CSA under flow conditions (Figure 4C) consistent with absence of PfEMP1 on the surface of parasite-infected host cells (Figure 4B). Additionally, the parasite lines CS2ΔMAL7P1.171, CS2ΔPF10_0025 and CS2ΔPF13_0076 showed greatly reduced levels of adherence which provides functional evidence of decreased levels of PfEMP1 on the infected erythrocyte surface (Figure 4B). Similar results were obtained using static adhesion assays to CSA (Figure S4). These results provide further evidence that the proteins encoded by PFB0106c, MAL7P1.172, PF14_0758, MAL7P1.171, PF10_0025 and PF13_0076 play a role in trafficking and display of PfEMP1 on the host cell surface (Figure 7).

**Figure 5:**
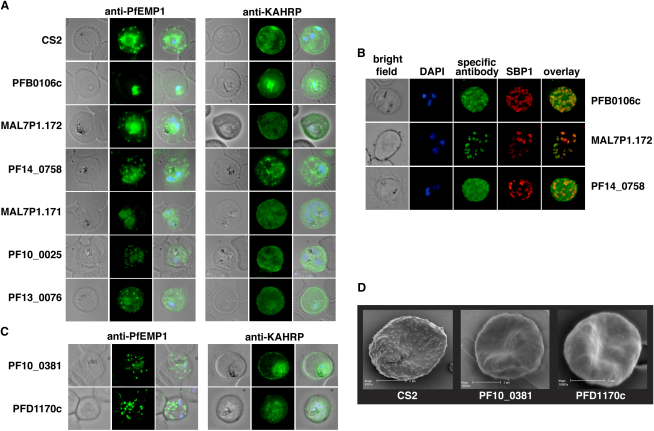
Microscopic Analysis of Mutants with Export Defects (A) Localization of PfEMP1 and KAHRP in mutant *P. falciparum*-infected erythrocytes. The parasite lines shown are those with either no PfEMP1 or reduced levels on the surface of infected erythrocytes determined by FACS and trypsin analysis. The first panel depicts localization of PfEMP1 and the second panel shows localization of KAHRP. The first column of each panel shows a bright-field image, the second panel specific antibody (either anti-PfEMP1 or anti-KAHRP) and the third panel overlay of the previous two and a nuclear stain (DAPI). (B) Localization pattern of three proteins which when deleted ablate surface exposure of PfEMP1. These proteins were detected with specific antibodies raised against the gene products of *PFB0106c, MAL7P1.172* and *PF14_0758* (see Figure 2C). The first panel shows a bright-field image, followed by a DAPI image, then the specific antibody (green), then antibodies against the Maurer's cleft resident protein SBP1 (red) and an overlay of the specific antibody with SBP1 localization. (C) Localization of PfEMP1 (first panel) and KAHRP (second panel) for parasite lines CS2ΔPF10_0381 and CS2ΔPFD1170c. Shown are a brightfield image, specific antibody (either anti-PfEMP1 or anti-KAHRP) and an overlay of the two with a nuclear stain (DAPI). (D) Scanning electron microscopy of CS2ΔPF10_0381 and CS2ΔPFD1170c infected erythrocytes. The first panel shows parental CS2-infected erythrocytes with normal knobs compared to the two mutant lines in which knobs are absent (PFD1170c) or greatly reduced in size (PF10_0381). The scale bar represents 2 μm.

To determine if loss of function in mutant parasite lines had an effect on distribution of PfEMP1, KAHRP, PfEMP3 or SBP1 in host erythrocytes we performed immunofluorescence experiments with antibodies (Figures 5, S5, and S7). None of the cell lines showed any trafficking defects of PfEMP3 or SBP1 (Figures S5 and S7). CS2ΔPFB0106c and CS2ΔMAL7P1.171 infected erythrocytes showed normal KAHRP distribution; however, PfEMP1 was primarily concentrated in the parasite with little detected within infected-erythrocytes (Figure 5A) suggesting the defect was a decreased efficiency of transfer of PfEMP1 to Maurer's clefts. PFB0106c protein in parental CS2-infected erythrocytes was distributed in the erythrocyte cytoplasm as well as localized to Maurer's clefts (Figure 5B) suggesting it is exported to the erythrocyte cytoplasm and interacts with Maurer's clefts as has been reported for KAHRP and PfEMP3 (Knuepfer et al., 2005; Wickham et al., 2001). Localization of PFB0106c protein to Maurer's clefts and the fact that PfEMP1 trafficking is blocked early within the parasite suggests this protein plays a role in transfer of this virulence protein to Maurer's clefts. The proteins MAL7P1.171 and PF10_0025 are likely to play a similar role; however, some PfEMP1 can be trafficked to Maurer's clefts and the infected erythrocyte surface by the mutant parasite suggesting they have an overlapping function with other protein(s) (Figure 7).

In contrast, PfEMP1 in both CS2ΔMAL7P1.172 and CS2ΔPF14_0758 infected erythrocytes showed localization to Maurer's clefts (Figure 5A), but not on the surface of infected erythrocytes (Figure 4B). The MAL7P1.172 protein seems to be mainly localized on Maurer's clefts in parental CS2-infected erythrocytes (Figure 5B and Movie S1). The movie in Fig. S8 shows that the protein appears to be localized within the lumen of the Maurer's cleft and is always surrounded by the membrane bound Maurer's clefts resident protein SBP1. In contrast, the PF14_0758 protein is distributed throughout the cytoplasm of infected erythrocytes with no major concentration on Maurer's clefts in parental CS2-infected erythrocytes (Figure 5B). Both of these mutant cell lines show a similar distribution of PfEMP1 to the CS2 parental line (Figure 5A). The mutant parasite line CS2ΔPF13_0076 also showed a normal distribution of PfEMP1 in the infected erythrocyte suggesting that any effect on trafficking of PfEMP1 is occurring at transfer from Maurer's clefts to the erythrocyte membrane. Overall these results identified exported proteins playing a role in trafficking of PfEMP1 to the host erythrocyte and provided evidence these proteins function at specific points in the pathway of trafficking (Figure 7).

### PFD1170c and PF10_0381 Are Required for Formation of Knobs

Two mutant parasite lines CS2ΔPFD1170c and CS2ΔPF10_0381 had reduced binding to CSA under static (Fig. S4) and flow conditions (Figure 4C). Interestingly, both lines expressed wild-type levels of var2csa PfEMP1 (Figure 4A). Additionally, transport of PfEMP1 to the erythrocyte surface was normal as measured by sensitivity of the exposed ectodomain to trypsin (Figure 4B) (Waterkeyn et al., 2000). Such behavior has previously been reported in knob negative parasites in which the major structural component of knobs, the *KAHRP* gene, had been disrupted (Crabb et al., 1997). We therefore determined the subcellular localization of PfEMP1 and KAHRP in CS2ΔPFD1170c and CS2ΔPF10_0381 (Figure 5C). The CS2ΔPF10_0381 infected erythrocytes showed similar localization of PfEMP1 as seen in the parent CS2 consistent with normal expression of this protein on the surface of host cells. KAHRP appeared to be in more localized punctate collections in CSΔPF10_0381 compared to the more uniform pattern observed in parental parasites. In contrast, CS2ΔPFD1170c-infected erythrocytes did not show the typical rim fluorescence when compared to parental cells suggesting a defect in movement of KAHRP from Maurer's clefts to the underside of the erythrocyte and assembly of the knob structure (Figure 7).

Knob morphology was examined by scanning electron microscopy in the two mutant lines (Figure 5D). Both CS2ΔPF10_0381 and CS2ΔPFD1170c parasite-infected red cells displayed dramatically altered knob morphology. CS2ΔPFD1170c showed a lack of knobs on the surface of infected red blood cells despite the fact that KAHRP was expressed and exported to the host erythrocyte. In contrast, erythrocytes parasitized with CS2ΔPF10_0381 had rudimentary knobs, which were significantly smaller and less protrusive compared to wild-type knobs (Fig. S6). Therefore the proteins encoded by *PFD1170c* and *PF10_0381* are required for knob formation in *P. falciparum*-infected erythrocytes (Figure 7).

### Identification of Genes that Affect Deformability of *P. falciparum*-Infected Erythrocytes

**Figure 6:**
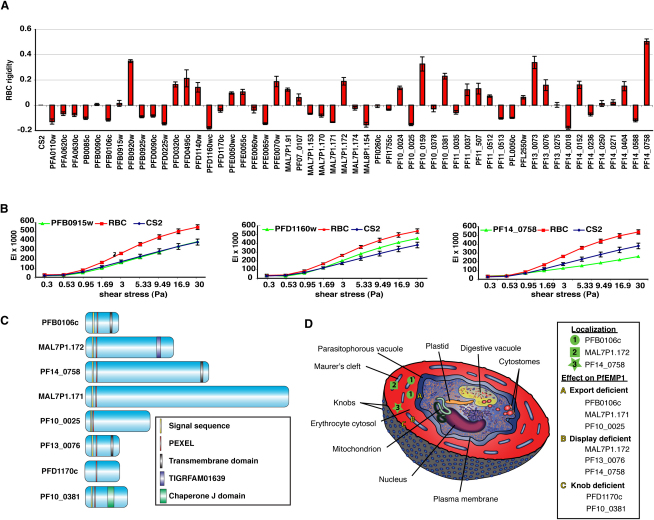
Genes Involved in *P. falciparum*-Infected Erythrocyte Rigidity and Properties of Proteins that Play a Role in Trafficking or Function of PfEMP1 (A) Rigidity as measured using the LORCA for all generated mutants compared to CS2. The four highest shear stress points (see Figure 5B) for each cell line was used to calculate the deformability ratio and compared to the ratio of CS2. (B) Examples of LORCA measurements comparing membrane rigidity of *P. falciparum*-infected erythrocytes. The erythrocyte rigidity (expressed as elongation index [EI]) conferred on the host cell by each mutant *P. falciparum* line (green) compared to parent CS2 (blue) and uninfected red cells (red) at increasing shear stress measured in pascal (Pa). Parasites were synchronised and concentrated to 40% parasitaemia to increase sensitivity of the measurement. Error bars indicate standard deviation. (C) Structure of the proteins that play a role in trafficking and function of PfEMP1. The gene number is shown for each protein from PlasmoDB (www.plasmodb). Yellow refers to a proposed signal sequence while red signifies the presence of a PEXEL required for export. Black shading corresponds to a proposed transmembrane region. Green refers to a DnaJ domain and blue a TIGRFAM01639 domain. (D) Diagrammatic representation of a *P. falciparum*-infected erythrocyte signifying the localization of the protein (green symbols) or their functional position with respect to effects on PfEMP1 trafficking when disrupted (yellow letters).

Upon infection with *P. falciparum,* erythrocytes become rigid, most likely due to export of parasite-derived proteins and cross-linking with the red blood cell cytoskeleton (Cooke et al., 2001). To determine if proteins encoded by the targeted genes have any influence on erythrocyte membrane rigidity, we assessed the deformability of infected red blood cells with a laser-assisted optical rotational cell analyzer (LORCA) (Hardeman et al., 1998) (Figures 6A and 6B). The deformability ratio of erythrocytes infected with wild-type parasite to erythrocytes infected with mutant parasites for the four highest shear stresses was calculated and plotted to compare the influence of the deleted protein on the rigidity of the infected erythrocyte (Figure 6A). The average ratio for uninfected erythrocytes was 0.67. Many of the mutant lines demonstrated small alterations in rigidity of the infected erythrocyte suggesting a large number of proteins potentially have a minor effect on this host cell property.

**Figure 7:**
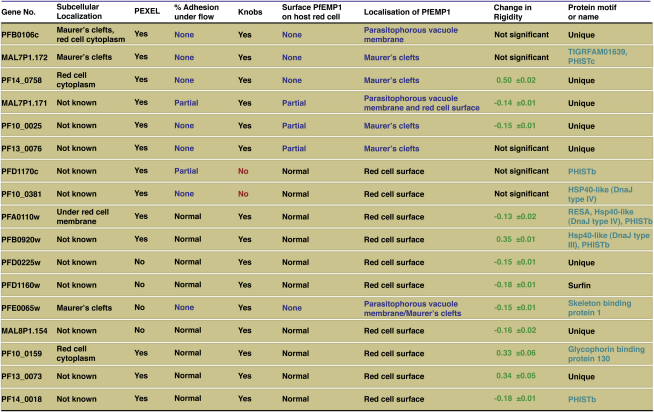
Function and Properties of Selected Proteins Identified by the *P. falciparum* Gene Knockout Screen These proteins play a role in function or trafficking of the major virulence protein PfEMP1 or a putative role in determination of rigidity. The full description of the genes and proteins can be found at http://plasmodb.org/. Most proteins have a PEXEL, which is required for export to the infected erythrocyte. Changes in rigidity conferred on the host cell by each mutant *P. falciparum* line was compared to parent CS2 and uninfected red cells at increasing shear stress and are relative to CS2-infected erythrocytes. Those shown as a positive number have a significantly increased rigidity whilst those that are negative have a decrease in rigidity that is more than the CS2ΔPFA0110w (RESA) parasite line that has been shown previously to have an altered rigidity (Silva et al., 2005). The TIGR FAM01639 domain in MAL7P1.172 represents a conserved sequence of about 60 amino acids found in over 40 predicted proteins of *P. falciparum*. It is not found elsewhere, including closely related species such as *P. yoelii*. The PHIST proteins share a homologous domain unique to Plasmodium of 150 amino acids and have been divided into a number of subfamilies (a, b, and c) (Sargeant et al., 2006). The skeleton binding protein 1 is included as a reference protein that is involved in PfEMP1 trafficking (Maier et al., 2007; Cooke et al., 2006).

However a number of mutant cell lines had a significantly reduced level of rigidity and we used CS2ΔPFA0110w as the cut off for significance (−0.13 ± 0.02) compared to CS2 as disruption of the *RESA* gene has been shown previously to affect rigidity (Silva et al., 2005) (Figure 7). Four cell lines CS2ΔPFB0920w, CS2ΔPF10_0159, CS2ΔPF13_0073 and CS2ΔPF14_0758 showed a significant increase in membrane rigidity (Figure 6A, Figure 7). Interestingly, CS2ΔPFB0920w, CS2ΔPF10_0159 and CS2ΔPF13_0073 were also high binders in the CS2 adhesion assay (Figure 4C) and in contrast, CS2ΔPF14_0758 lacked erythrocyte surface PfEMP1 (Figure 4B). These results suggest that a number of *P. falciparum* proteins combine to determine the overall rigidity of the parasite-infected erythrocyte (Figure 7).

## Discussion

The *P. falciparum*-infected erythrocyte undergoes a series of modifications after invasion converting a terminally differentiated cell into one in which the parasite can access nutrients and grow within a niche relatively protected from host responses. The mediators responsible for remodeling the erythrocyte are most likely exported proteins (Sargeant et al., 2006). However, there is information on specific roles for only a handful of these proteins. In order to address the function of exported proteins we used a gene knockout strategy combined with functional assays. Using this approach we identified exported proteins required for trafficking, display and function of the cytoadherence protein PfEMP1, assembly of knobs and rigidification of the infected red cell, properties that are all thought to be important in malaria pathogenesis (Figure 7).

The virulence protein PfEMP1 is expressed early post invasion; however, it does not appear on the *P. falciparum*-infected erythrocyte surface until 16 hr after invasion when the host cells become adherent (Kriek et al., 2003). The mechanism and proteins required for trafficking of PfEMP1 through the parasitophorous vacuole membrane into Maurer's clefts and to the erythrocyte membrane are unknown. In this study we have identified six proteins that have an effect on normal trafficking of PfEMP1 (Figures 6C, 6D, and 7). Disruption of function for PFB0106c, MAL7P1.172, PF14_0758, and PF13_0076 resulted in a complete lack or greatly reduced levels of PfEMP1 on the parasite-infected erythrocyte suggesting they are required for subcellular localization of this virulence protein. Trafficking of other exported proteins such as the classical PEXEL-containing proteins KAHRP and PfEMP3 (Figures 5A and S5) and the non-PEXEL containing exported protein SBP1 (Figure S5) is not affected suggesting that the proteins identified are specifically required for localization of PfEMP1.

The gene products of PFB0106c, MAL7P1.171 and PF10_0025 seem to interfere with early steps of PfEMP1 transport, since less PfEMP1 is detected in erythrocytes infected with parasites deficient of these molecules. In parasite lines deficient in either MAL7P1.172, PF14_0758 or PF13_0076, PfEMP1 was trafficked to Maurer's clefts suggesting the function of the relevant proteins is in transfer from this parasite structure to the erythrocyte membrane (Figure 6D). Previous studies have identified SBP1 as functioning at or just prior to this point (Cooke et al., 2006; Maier et al., 2007) and additional molecular players in this step are now revealed. The precise interplay between these proteins will require further studies. In contrast, PfEMP1 in CS2ΔPFB0106c does not appear to be transferred to Maurer's clefts suggesting this protein functions early when PfEMP1 is loaded into these structures (Figure 6D). Interestingly, in CS2ΔMAL7P1.172 parasites PfEMP1 was not readily detected on Western blots using the standard solubilization procedure for this protein. One explanation could be that PfEMP1 in this line has different solubility characteristics perhaps due to its blockage at Maurer's clefts in its trafficking route. This is consistent with previous data showing that the solubility of PfEMP1 changes during its transport pathway (Papakrivos et al., 2005). Consistent with the hypothesis that expansion of the exportome in *P. falciparum* is primarily for trafficking and function of PfEMP1 and human specific pathogenicity mechanisms is the observation that the identified molecules are either *P. falciparum* specific or found exclusively in the other *Plasmodia* of primates.

Our screen revealed that disruption of PFD1170c and PF10_0381 protein function leads to absent or greatly decreased knob structures with an abnormal distribution. These same disruptants also showed reduced cytoadherence providing a phenotype similar to that observed for KAHRP disruption (Crabb et al., 1997) (Figure 4C) and suggesting the proteins encoded by these genes are required for correct assembly of KAHRP into knobs (Figures 6D and 7). Interestingly, when *P. falciparum* isolates with different adhesion properties were compared in a proteomic analysis, PFD1170c was identified as being expressed at 3-fold increased levels in the membrane of infected erythrocytes of different strains (Florens et al., 2004). In light of our results, it is plausible that the increased expression of the PFD1170c protein results in a higher density of knob structures and therefore increased adherence.

An interesting family of proteins that are exported in *P. falciparum* are the DnaJ proteins and these are likely to function as co-chaperones with HSP70 to fold and assemble protein structures within the parasite-infected erythrocyte. Eleven of these were not essential for in vitro growth and are likely to be involved in overlapping functions. Interestingly, three of the genes with DnaJ domains could not be disrupted and presumably are involved in essential functions. One is a DnaJ type I protein and conserved across all *Plasmodium spp*. and is likely to be required as a cochaperone for a conserved set of protein(s). The PF10_0381 protein has a DnaJ domain and is classified as HSP40-like, providing a clue to its function in knob assembly (Figure 6D). Recently, it has been suggested that the type III class of Hsp40 proteins should be divided into a new type IV class that exhibit variations in the HDP catalytic motif within the conserved J domain (Botha et al., 2007) and PF10_0381 can be classified in this group. In general Hsp40 proteins can serve two roles; first, targeting protein substrates to Hsp70 for folding and second, stabilization of Hsp70 in a substrate-bound form. However, as yet type III and IV Hsp40 proteins have not been shown to bind polypeptide substrates and it has been suggested they may not have chaperone activity. They may serve a specialized role in recruitment of Hsp70 for folding of specific substrates and PF10_0381 may play a direct role in assembly of KAHRP within knob structures.

Severe malaria caused by *P. falciparum* can involve multiple organ failure and this is associated with increased rigidity of parasite-infected erythrocytes that can contribute to blockage of micro-capillaries (Nash et al., 1989). Normal erythrocytes are highly deformable allowing them to flow through the smallest capillaries and this property is due to their low internal viscosity, high-surface-area to volume ratio, and the elastic nature of the erythrocyte membrane and underlying cytoskeleton. As the *P. falciparum* parasite grows within the erythrocyte it loses its deformability and becomes spherocytic and more rigid (Cooke et al., 2004). These properties are thought to contribute to the pathogenesis of malaria, in addition to vascular adhesion of parasitised erythrocytes. The altered deformability is manifested by export of proteins into erythrocytes that interact with the host cell cytoskeleton and insert into the membrane. Using micro-pipetting techniques it has been shown KAHRP and PfEMP3 contribute to altered membrane rigidity of *P. falciparum*-infected erythrocytes (Cooke et al., 2006). In this study, it was not possible to use micropipetting methods on such a large number of mutant cell lines and we therefore used LORCA, which has allowed a higher throughput analysis (Hardeman et al., 1998). However, the LORCA has disadvantages in that the sensitivity is not the same as micro-pipetting and as a result we may have missed identifying some mutant lines with rigidity phenotypes. Nevertheless, it was clear that a number of mutant cell lines had altered erythrocyte rigidity compared to the parental line, suggesting that a large number of exported proteins contribute to the overall rigidity of the erythrocyte.

It is interesting that in *S. cerevisiae* 19% of genes are essential and under experimental conditions the functions of most are not required (Giaever et al., 2002). In *P. falciparum*, at least for the gene set we have chosen, 36% appeared to be essential suggesting that there may be less redundancy in function for this protozoan parasite (Figure 3A). However, this figure may be somewhat high due to the fact that genetic tools in this parasite are not as well developed and therefore efficiency of targeting may be less optimal (Maier et al., 2006). However, it is clear that the genes encoding exported proteins are generally dispensable for in vitro growth with only 23.5% of these apparently essential using current genetic tools.

The exported proteome is predicted to comprise 455 proteins (∼8% of the genome) and of these, 256 code for the variant proteins PfEMP1 (59), stevor (32) and rifins (165) (Sargeant et al., 2006). The remaining 199 consist of unique genes and a number of gene families that, for example, encode proteins that have a DnaJ or a PHIST domain. The reasons for the greatly expanded exported proteome in *P. falciparum* are not clear, however, this organism is unique in its expression of PfEMP1. We have suggested previously that a proportion of the exported proteins would be required for trafficking and function of this complex protein (Sargeant et al., 2006). Consistent with this hypothesis is the identification of eight genes that encode proteins involved in either PfEMP1 function or act as ancillary proteins required for assembly of knobs. It is likely that there will be other genes involved in these functions that are yet to be identified. Additionally, many proteins may have overlapping functions and this redundancy would not be detected in our gene knockout screen.

A core set of 36 exported proteins has been defined that are conserved in the genus *Plasmodium*, i.e., they can be found in at least two *Plasmodium* species (Sargeant et al., 2006). We were able to disrupt 7 out of 9 attempted core set genes, all of which are specific to the primate lineage (Figure 3D). One exception is PFD0495c, which is one of only two core molecules where orthologs can be found in all primate and mouse malaria parasites examined (Sargeant et al., 2006). The fact that a majority of this core set is dispensable is rather surprising, since a broader distribution of genes within the genus could implicate a more fundamental importance. Interestingly, three of the gene deletions resulted in PfEMP1 transport defects (*MAL7P1.172, PF10_0025* and *PF13_0076*). Since PfEMP1 does not have any orthologs in most other *Plasmodium* species the proteins encoded by these genes are likely to be involved in trafficking and export of a number of proteins.

In summary, we have used a gene knockout approach on a scale not previously attempted in this organism to address the role of *P. falciparum* proteins that are exported into the parasite-infected erythrocyte. Collectively these proteins act like the secretion systems seen in bacteria in which pathogenicity arises from secreted proteins that interact with host cells by direct injection or by their presence in the extracellular milieu (Abdallah et al., 2007). The complexity of the secreted protein repertoire and the number of membranes that must be crossed make the *Plasmodium* secretion system more comparable to secretion systems of Gram negative bacteria (Christie et al., 2005; Cornelis and Van Gijsegem, 2000), however they appear significantly more complex due to the reconstitution of a protein trafficking system within the red cell and involvement of multiple chaperone molecules. Although it may not be worthwhile labeling them as a Type VIII secretion system, it may be valuable to adopt approaches being tested in bacteria in which these systems are the target of new therapeutic approaches aimed at minimizing pathogen virulence (Cegelski et al., 2008). This study significantly extends our understanding the role of exported proteins in host/parasite interactions essential for survival of *P. falciparum* in vivo and defines a group of potentially novel therapeutic targets.

## Experimental Procedures

### Culture, Parasite Strains, Plasmid Constructs and Immunoblots

CS2 wild-type parasites, a clone of the It isolate, adheres to chondroitin sulfate A (CSA) and hyaluronic acid in vitro. Constructs were assembled in pHHT-TK (Duraisingh et al., 2002) or pCC1 (Maier et al., 2006) and transfected as described (Crabb et al., 1997). To generate antibodies either GST-fusion proteins or KLH-coupled fusion peptides were synthesized (Invitrogen) and injected into rabbits and IgG purified. Immunoblots were performed as described (Maier et al., 2007).

### Adherence and Trypsin Cleavage Assays

Adherence assays under static and flow conditions to CSA were performed using *P. falciparum* –infected erythrocytes at 3% parasitemia and 1% hematocrit (Crabb et al., 1997). For trypsin cleavage, trophozoite stage parasites were either incubated in TPCK-treated trypsin (Sigma) (1 mg/ml in PBS), in PBS alone or in trypsin plus soybean trypsin inhibitor (5 mg/ml in PBS, Worthington, Lakewood, NJ, USA) at 37°C for 1 hr and analyzed as described (Waterkeyn et al., 2000).

### Laser-Assisted Optical Rotational Cell Analysis

To measure deformability the infected red cells were analyzed via a laser-assisted optical rotational cell analyzer (LORCA). Two independent measurements were taken and repeated in independent experiments. Each experiment included measurement of CS2 cultured in an identical red cell batch and uninfected erythrocytes (see supplementary procedures).

### Electron Microscopy and Immunofluorescence Microscopy

For scanning electron microscopy parasite-infected red blood cells were tightly synchronised and processed as described (Rug et al., 2006). For immunofluoresence analysis, acetone/methanol (90%/10%) fixed smears of asynchronous parasites of CS2Δ- and/or CS2WT-infected erythrocytes were probed with rabbit anti-ATS (1:50), preabsorbed mouse anti-ATS (1:50), rabbit anti-KAHRP (1:200), mouse anti-KAHRP (His; 1:50), rabbit anti-SBP1 (1:500), mouse anti-SBP1 (1:500), mouse anti-PfEMP3 (1:2000), rabbit anti-PfEMP3 (1:1000), rabbit anti-PF14_0758 (1:125), rabbit anti-MAL7P1.172 (1:250), rabbit anti-PFB0106c (1:50) and consequently incubated with Alexa Fluor 488 conjugated anti-rabbit IgG (Molecular Probes) and Alexa Fluor 488 conjugated anti-mouse IgG (Molecular Probes). See Supplemental Experimental Procedures for more detail.

### Antibodies to the Surface of *P. falciparum* Infected Erythrocytes

Serum samples were tested for IgG binding to the surface of trophozoite-infected erythrocytes at 3%–4% parasitemia, 0.2% hematocrit, using flow cytometry, as described (Beeson et al., 2006). All samples were tested in duplicate. Sera were collected from malaria-exposed pregnant residents of Madang Province, Papua New Guinea, presenting for routine antenatal care at the Modilon Hospital, Madang (Beeson et al., 2007). Written informed consent was given by donors and ethical clearance obtained from the Medical Research Advisory Committee, Department of Health, PNG, and Walter and Eliza Hall Institute Ethics Committee. Serum samples collected from Melbourne residents were used as controls.

## Supplemental Data

Document S1. Supplemental Experimental Procedures, Nineteen Figures, Three Tables, and Supplemental References

Movie S1. Movie of the MAL7P1.172 Protein that upon Deletion Showed Trafficking Defects of PfEMP1 MAL7P1.172 (green) is localised in the lumen of Maurer's clefts surrounded by the membrane protein SBP1 (red).
