# Supplementary material for: Exported Proteins Required for Virulence and Rigidity of Plasmodium falciparum-Infected Human Erythrocytes
Source: Cell. 2008 Jul 11;134(1):48–61. doi: 10.1016/j.cell.2008.04.051 (PMC2568870; doi:10.1016/j.cell.2008.04.051)

Cell, Volume *134*

Supplemental Data

Exported Proteins Required for Virulence and Rigidity of *Plasmodium falciparum*-Infected Human Erythrocytes

Alexander G Maier, Melanie Rug, Matthew T O'Neill, Monica Brown, Srabasti Chakravorty, Tadge Szestak, Joanne Chesson, Yang Wu, Katie Hughes, Ross L Coppel, Chris Newbold, James G Beeson, Alister Craig, Brendan S Crabb, and Alan F Cowman

Supplemental Experimental Procedures

Culture conditions and parasite strain.

Erythrocytic stages of *P. falciparum* were maintained in human 0+ erythrocytes (Crabb et al., 1997). CS2 wild-type parasites, a clone of the It isolate (Rogerson et al., 1995), adheres to chondroitin sulphate A (CSA) and hyaluronic acid *in vitro*. Parasites were selected for the adherence to bovine trachea CSA (Sigma, ST Louis, MO, USA) prior to transfection.

Plasmid constructs, transfection and Southern blotting.

Constructs were either assembled in pHHT-TK (Duraisingh et al., 2002) or pCC1 (Maier et al., 2006) vectors (see below). The vectors contain a hDHFR cassette (driven by a calmodulin promoter) flanked by 2 multiple cloning sites to accept targeting sequences of the relevant gene. They also include a negative selection cassette (driven by the Hsp86 promoter region) to select parasites in which double recombination events had occurred. Plasmid DNA was extracted using Maxiprep kits from either Qiagen or Invitrogen (Purelink). 80 μg DNA was tranfected for each transgenic line using standard protocols (Crabb et al., 1997). After positive selection on WR99210 the cells were placed under negative selection using either Ganciclovir (Roche, 20μM, pHHT-TK) or 5-Fluorocytosine (ICN, 100 nM, pCC1). If no cells were recovered the negative selection was repeated at least twice. Any resulting cell populations underwent Southern blot analysis. Genomic DNA was prepared with the Dneasy Tissue Kit (Qiagen) and Southern Blot analysis performed using the DIG system (Roche) according to manufacturer's instructions to confirm disruption of the targeted genes.

Primer sequences used for the construction of pCC1:

Aw132 ctagagtagatctgtcttaaggtggatccgtaagcttgtgaattcgtgagct

Aw133 cacgaattcacaagcttacggatccaccttaagacagatctact

Aw118 atcggatcctttttatggaagacgccaaaaacataaagaaaggcccgg

Aw119 gatgataagcttacacggcgatctttccgccc

Aw76 caatggccccttttcttaagcattttg

Aw77 gcatggatcctgatatatttctattagg

Aw122 atccccccgggggtaccctgcaggtcgacttaattaaggatatggcagcttaatgttcgtttttc

Aw123 tactactagcggccgcctaccctgaagaag

aw500 atcctcgagatggtgacagggggaatg

Aw501 ggatcccgggttaaacacagtagta

In assembling pCC1 the goal was to construct a modular vector with gene cassettes for negative and positive selection and multiple cloning sites for the incorporation of gene-specific targeting sequences. We modified pGEM7Z(+) (Promega) by annealing a polylinker consisting of oligonucleotides aw132 and aw133 into the XbaI/SacI sites of pGEM7Z(+). This yielded the cloning vector LT-1. We then amplified firefly luciferase from pPf86 (kindly provided by Kevin Militello, Harvard School of Public Health) with the primer pair aw118/119 and cloned it into the *Bam*H I/*Hind* III sites of LT-1 creating the vector LT-2. The 3' UTR of the gene encoding the histidine rich protein 2 (HRP2 3') was cut out of the vector pHHT-TK (Duraisingh et al., 2002) with *Hind* III/*Eco*R I and annealed into LT-2. The 5' UTR of the *P. falciparum* calmodulin (CAM) gene was amplified with the primer pair aw76/77 from pHHT-TK and ligated into the HRP2 3' containing LT-2. This plasmid was named LT-3.

The 3' UTR of the *P.berghei* dihydrofolate reductase/thymidylate synthase (*PbDT* 3') was amplified with the oligonucleotides aw122/123 and ligated into the *Not* I/*Xma* I cut plasmid pHHT-TK resulting in pHHT-TK-3'. The firefly luciferase was cut out of LT-3 with *Bam*H I/*Hind* III and replaced with the human dihydrofolate reductase gene (hDHFR) from pHHT-TK. The *hDHFR* containing LT-3 was then cut with *Eco*R I/*Afl* II to release the whole *hDHFR* gene cassette (with the *CAM5'* and *HRP2* 3') and cloned into *Eco*R I/*Afl* II cut pHHT-TK-3'.

The resulting vector was named pDC1 and contains a *CAM*5'-*hDHFR*-*HRP2* 3' gene cassette for positive selection and a *HSP86* 5'-*Herpex simplex TK*-*PbDT* 3' gene cassette for negative selection. The component of each gene cassette can be individually cut out and replaced (hence the vector is modular). Each gene cassette is flanked by a multiple cloning site. In addition the vector contains a plasmid backbone, which enables ampicillin selection in *E. coli* and replication both in *E.coli* and *P.falciparum*. Finally the *ScCDUP* gene was amplified with the primers aw500 and aw 501 from the plasmid pHHT-CDUP-ΔPF11_0037 (Maier et al., 2006) and cloned *Xho* I/*Xma* I into the cut pDC-1 to replace the *HsTK* gene with the *ScCDUP* gene. The final vector was then called pCC-1.

Generation of antibodies.

The following KLH-coupled fusion peptides were synthesised (Invitrogen) and injected into rabbits: PFB0106c, amino acid 164-177, R878; MAL7P1.172, amino acid 117-130, R883; PF14_0758, amino acid 780-793; R884; PFE0060w, amino acid 128-147, R679; PF10_0159, amino acid 808-821, KW51-1; PF13_0275, amino acid 266-279, KW59-2. GST-fusion proteins for the genes PF14_0018 (amino acid 217-

304; R688), PF11_0037 (amino acid 150-216; R687) were expressed and rabbits immunized. IgG from the rabbit sera were purified via a protein G sepharose column and eluted with 100 mM glycine-HCl, pH 2.5, dialysed against PBS and their concentration adjusted to ~5 mg/ml.

SDS-PAGE (polyacrylamide gel electrophoresis) and immunoblot analysis.

Synchronised trophozoite cultures were saponin lysed, the pellet was washed 3 times in PBS and taken up in reducing SDS sample buffer (Invitrogen). Proteins were separated on 3-8% Tris-Acetate or 10% Bis-Tris gels (Invitrogen, Carlsbad, CA, USA). Western blotting to nitrocellulose (0.45µm; Schleicher and Schuell, Dassel, Germany) was performed according to standard protocols. In addition to the already mentioned antibodies mouse monoclonal anti-ATS 1B/98-6H1-1 (1:200) (preabsorbed on erythrocyte ghosts) was used (Maier et al., 2007). Horseradish peroxidase-coupled sheep anti-rabbit Ig (1:1000) or anti-mouse Ig (1:2000) (Chemicon, Melbourne, Australia) were used as secondary antibodies.

CSA adherence assays.

Static binding assays (50 mg/ml) and binding under physiological flow conditions (100 mg/ml) to CSA were performed using *P. falciparum*–infected erythrocytes at 3% parasitemia and 1% hematocrit (Crabb et al., 1997). For panning purposes plastic petri dishes were coated with 100 µg/ml CSA overnight and blocked with 10% human serum in RPMI-HEPES. Synchronised cultures at the trophozoite stage were enriched via gelatine flotation and added to the CSA coated petri dishes, where adhesion was allowed to occur for 1 h at 37°C. Unbound cells were washed off with three washes with RPMI-HEPES, pH6.8. Bound cells were resuspended and taken into culture to

expand, before another round of selection commenced. After 3 rounds of selection the cultures were analysed for the expression of PfEMP1 via a trypsin cleavage assay.

Flow based cytoadherence assays.

Flow assays on protein-coated microslides were performed using standard conditions (Tse et al., 2004), with a coating concentration of CSA of 100 μg/ml. All cell lines were tested in duplicate in three separate experiments. The results are expressed as number of bound infected red blood cells per mm^2. Cell lines displaying binding values outside of the 95% confidence interval of the CS2 parental line were regarded as having a different binding phenotype.

Trypsin cleavage assays.

For trypsin cleavage sorbitol synchronised parasites were grown to trophozoite stage and enriched via gelatine [Gelofusine, Braun, Bella Vista, Australia] flotation. Infected red blood cells were then either incubated in TPCK-treated trypsin (Sigma) (1 mg/ml in PBS), in PBS alone or in trypsin plus soybean trypsin inhibitor (5mg/ml in PBS, Worthington, Lakewood, NJ, USA) at 37°C for 1h. Trypsin inhibitor was then added to the trypsin and PBS aliquot to be incubated at room temperature for 10 min (Waterkeyn et al., 2000). Cell pellets were extracted in the presence of protease inhibitors (Complete, Roche) with Triton X-100 (1%) and subsequently with sodium dodecylsulfate (SDS, 2%) as previously described (Baruch et al., 1996).

Laser-assisted optical rotational cell analysis.

To measure deformability the infected red blood cells were subjected to analysis via a laser-assisted optical rotational cell analyser (LORCA). In this assay erythrocytes are taken up in a polymer solution sitting in a gap between an inner cylinder and an outer

cup. By rotating the cup shear stress is created, which in turn forces the erythrocytes to change from a biconcave to an ellipsoid morphology. The change in morphology can be detected via changes in the diffraction pattern created by a laser-beam shining through the solution. The amount of shear stress applied is regulated by the speed of the spinning cup and each observed value per measurement is the equivalent of 25,000 cells. Synchronised cultures with >4% parasitemia at trophozoite stage were enriched via gelatine flotation, washed twice in RPMI-HEPES buffer and then adjusted to a final parasitemia of 40% and 50% hematocrit with uninfected gelatine mock-treated red blood cells. 25µl of this mixture was added to 5ml of ~50g/l polyvinylpyrrolidone (PVP) in PBS pH7.4 (final viscosity 30 ±2 mPa*s at 37°C) and measurements were taken at shear stresses between 0 and 30 Pa at 37°C. Two measurements were taken and repeated in an independent set of experiments. Each set of experiments included the measurement of CS2 wild-type cells and uninfected red blood cells. They were cultured in an identical red cell batch.

Immunofluorescence microscopy.

For immunofluoresence analysis, acetone/methanol (90%/10%) fixed smears of asynchronous parasites of CS2Δ- and/or CS2WT-infected erythrocytes were probed with rabbit anti-ATS (1:50), preabsorbed mouse anti-ATS (1:50), rabbit anti-ATS (1:50), rabbit anti-KAHRP (1:200), mouse anti-KAHRP (1:50), rabbit anti-SBP1 (1:500), mouse anti-SBP1 (1:500), mouse anti-PfEMP3 (1:2000), rabbit anti-PfEMP3 (1:1000), rabbit anti-PF14_0758 (1:125), rabbit anti-MAL7P1.172 (1:250), rabbit anti-PFB0106c (1:50) and consequently incubated with secondary antibodies Alexa Fluor 488 conjugated anti-rabbit IgG (Molecular Probes) and Alexa Fluor 488 conjugated anti-mouse IgG (Molecular Probes). To avoid photobleaching cells were

covered with Vectashield (Vector Laboratories, Burlingame, CA) containing 0.2 ng/µl DAPI (Roche) to stain for parasite DNA. Rabbit and mouse antibodies against the same antigen were used to verify the pattern observed in each cell line and only one representative of each is shown in the figure (Fig. 4 and Fig. S7). Cells were viewed with a Plan-Neofluar 100x/1.3 oil objective on a Zeiss Axiovert 200M Live Cell Imaging Inverted Microscope equipped with a AxioCam MRm camera and primarily processed with AxioVision 4.4 deconvolution software package. Captured images were then further processed using Photoshop and ImageJ software (available from http://rsb.info.nih.gov/ij). Pictures were adjusted to gain optimal contrast to visualize features of interest. For the supplementary data movies, cells were treated as described above and viewed with an HCX PL APO 100x/1.4NA objective on a Leica SP5 Spectral Confocal Microscope and the z-stacks were processed using the Leica LAS Advanced Fluorescence software package.

Antibodies to the surface of *P. falciparum* infected erythrocytes.

Serum samples were tested for specific IgG to the surface of pigmented trophozoite-infected erythrocytes at 3-4% parasitemia, 0.2% hematocrit, using flow cytometry, as described (Duffy et al., 2005). Cells were sequentially incubated with test serum diluted 1/20, rabbit anti-human IgG (Fc-specific, Dako; 1:100), and Alexa-Fluor-488-conjugated anti-rabbit Ig (Molecular Probes; 1:1000), with ethidium bromide 10 µg/ml in darkness. Incubations were 30 min each, performed at room temperature. Samples were analysed using a FACSCalibur flow cytometer (Becton-Dickinson, USA) and Flowjo software (TreeStar, USA). Fluorescence in channel FL1 was used as a measure of IgG binding and for each sample the geometric mean fluorescence of

uninfected red blood cells was deducted from the geometric mean fluorescence of infected erythrocytes. All samples were tested in duplicate.

Serum samples.

Sera were collected from malaria-exposed pregnant residents of the Madang Province, Papua New Guinea (PNG), presenting for routine antenatal care at the Modilon Hospital, Madang. This population experiences year-round transmission of *P. falciparum*. Sera from non-malaria exposed Australian residents were included as controls. Written informed consent was given by all donors and ethical clearance was obtained from the Medical Research Advisory Committee, Department of Health, PNG, and the Walter and Eliza Hall Institute Ethics Committee.

Scanning Electron Microscopy.

Scanning electron microscopy (SEM) was performed with trophozoites (20-28 h) harvested by magnetic cell sorting (CS columns; Miltenyi Biotec) followed by glutaraldehyde fixation (2% in PBS, Electron Microscopy Sciences), for 30 minutes at room temperature. Cells were washed 3 times in PBS, transferred to polyethylinamine-coated coverslips (Sigma, St Louis, MO), immersed in 10% ethanol, and dehydrated (25%, 50%, 70%, 90%, 2x100%; 10 minutes each). Cells were subjected to critical point drying (CPD030; Bal-Tech), coated with platinum in a sputter coater (S150B Sputter Coater; Edwards), and viewed in a Philips XL30 FEG scanning electron microscope at 120 kV.

Supplemental Figures.

Figure S1.

Southern blot analysis for all obtained gene knock-outs.

Genomic DNA from CS2 and transfected cell lines was digested with indicated combinations of restriction enzymes and hybridised with the 5' or 3' targeting region of the deleted gene. Expected sizes for wild-type (WT) locus (3D7 strain), for the locus with integration of the *hDHFR* cassette via double recombination and for the plasmid are indicated in kilobases (kb).

Figure S2.

Quantitative Southern blot for CS2ΔPFD0095c, CS2ΔMAL7P1.149, CS2ΔMAL8P1.153.

These 3 genes showed both wild-type and KO bands after negative selection and cloning by limited dilution. A possible explanation is the duplication of the gene locus to accommodate both the selection cassette and the maintenance of the expression of the gene. gDNA for parental cell lines (CS2) and 2 clones of the transgenic cell lines were digested by the indicated restriction enzymes. The subsequent Southern Blot was hybridised with a probe for the 5' targeting sequence. To ensure equal loading the Southern blot was stripped and reprobed with a probe against the single copy gene dihydropteroate synthase (*DHPS*). Sizes are indicated in kilobases (kb). Expected sizes are based on 3D7 sequence.

Figure S3.

Trypsin cleavage assay of upselected cell lines.

To ensure that the reduced binding of erythrocytes infected with the transgenic lines CS2ΔPFA0620c, CS2ΔPFB0090c and CS2ΔPFE0060w is due to a switch to the expression of another *var* gene these cultures were subjected to "panning" on CSA. After 3 rounds of selection these cell lines were referred to as PFA0620c up, PFB0090c up and PFE0060w up, respectively, and a trypsin cleavage assay was performed. The full-length PfEMP1 and the cytoplasmic tail were detected using antibodies to the acidic terminal segment (ATS) at the C-terminus of PfEMP1. The lanes for each parasite-infected red blood cell show: untreated (-), trypsin treated (+) and treated with trypsin and soybean trypsin inhibitor (i). As a comparison CS2 wild-type infected erythrocytes (CS2) (which express var2CSA PfEMP1) and uninfected red blood cells were subjected to trypsin cleavage too. Full-length var2CSA PfEMP1 and the two trypsin-resistant bands at 70 and 90 kDa are indicated by arrows. After 3 rounds of CSA panning the majority of cells had been selected for the expression of var2CSA, although (especially in erythrocytes infected with PFA0620c up and PFE0060w up) there was still a detectable subpopulation expressing another PfEMP1 as indicated by additional trypsin-resistant bands at 80-90kDa and additional full length bands of different sizes. However, these experiment show that the deletion of these genes are neither responsible for the switch in the PfEMP1 nor that this prevents the cells from reverting to the expression of var2CSA. In addition it shows that in these cells – independent from the *var* gene expressed – PfEMP1 is still exported to the surface of the infected red blood cell. Functionally erythrocytes infected with these CSA up-selected parasite lines display an increased ability to bind to CSA (Fig.

3C) and are being increasingly recognised by var2CSA specific antibodies by FACS assays (Fig. 3A).

Figure S4.

Adhesion assay under static conditions.

Adherence of each of the *P. falciparum* mutant strains was tested for its ability to bind to CSA under static conditions. The number of parasitised cells bound to surface coated with 50 µg/ml CSA was counted as bound infected red blood cells/mm^2. Shown is the mean of at least 2 independent experiments done in triplicates for each cell line. Values below the standard deviation of CS2 parental binding (mean = 281 bound infected red blood cell/mm^2) are depicted in green and values above are shown in red.

Figure S5.

Screen for transport defect of PfEMP3 and the Maurer's cleft marker SBP1 via immunofluorescence assay on cell lines deficient in the expression of molecules involved in PfEMP1 trafficking.

The first column in each panel shows a bright field image, the second the DAPI nuclear stain, the third the PfEMP3 (Waterkeyn et al., 2000) or SBP1 (Cooke et al., 2006; Maier et al., 2007) fluorescence and the fourth an overlay of the previous images. No major differences were observed in these cell lines.

Figure S6.

Scanning electron micrographs to detect knobs on surface of RBC infected with *P. falciparum* cell lines deficient in the expression of molecules involved in PfEMP1 trafficking.

One representative cell of >30 examined is shown.

Figure S7.

Immunofluorescence analysis of all mutant cell lines generated.

Mutants were screened for defects in PfEMP1, PfEMP3, KAHRP and SBP1 trafficking with no major differences observed. The first column in each panel shows a bright field image, the second the DAPI nuclear stain, the third PfEMP1 (Maier et al., 2007), KAHRP (Rug et al., 2006), PfEMP3 (Waterkeyn et al., 2000) or SBP1 (Cooke et al., 2006; Maier et al., 2007) fluorescence, respectively, and the fourth an overlay of the previous images.

SUPPLEMENTAL REFERENCES

Baruch, D.I., Gormley, J.A., Ma, C., Howard, R.J., and Pasloske, B.L. (1996). *Plasmodium falciparum* erythrocyte membrane protein 1 is a parasitized erythrocyte receptor for adherence to CD36, thrombospondin, and intercellular adhesion molecule 1. Proc Natl Acad Sci USA *93*, 3497-3502.

Cooke, B.M., Buckingham, D.W., Glenister, F.K., Fernandez, K.M., Bannister, L.H., Marti, M., Mohandas, N., and Coppel, R.L. (2006). A Maurer's cleft-associated protein is essential for expression of the major malaria virulence antigen on the surface of infected red blood cells. J Cell Biol *172*, 899-908.

Crabb, B.S., Cooke, B.M., Reeder, J.C., Waller, R.F., Caruana, S.R., Davern, K.M., Wickham, M.E., Brown, G.V., Coppel, R.L., and Cowman, A.F. (1997). Targeted gene disruption shows that knobs enable malaria-infected red cells to cytoadhere under physiological shear stress. Cell *89*, 287-296.

Duffy, M.F., Byrne, T.J., Elliott, S.R., Wilson, D.W., Rogerson, S.J., Beeson, J.G., Noviyanti, R., and Brown, G.V. (2005). Broad analysis reveals a consistent pattern of var gene transcription in *Plasmodium falciparum* repeatedly selected for a defined adhesion phenotype. Mol Microbiol *56*, 774-788.

Duraisingh, M.T., Triglia, T., and Cowman, A.F. (2002). Negative selection of *Plasmodium falciparum* reveals targeted gene deletion by double crossover recombination. Int J Parasitol *32*, 81-89.

Maier, A.G., Braks, J.A., Waters, A.P., and Cowman, A.F. (2006). Negative selection using yeast cytosine deaminase/uracil phosphoribosyl transferase in *Plasmodium falciparum* for targeted gene deletion by double crossover recombination. Molecular & Biochemical Parasitology *150*, 118-121.

Maier, A.G., Rug, M., O'Neill, M.T., Beeson, J.G., Marti, M., Reeder, J., and Cowman, A.F. (2007). Skeleton-binding protein 1 functions at the parasitophorous vacuole membrane to traffic PfEMP1 to the *Plasmodium falciparum*-infected erythrocyte surface. Blood *109*, 1289-1297.

Rogerson, S.J., Chaiyaroj, S.C., Ng, K., Reeder, J.C., and Brown, G.V. (1995). Chondroitin sulfate A is a cell surface receptor for *Plasmodium falciparum*-infected erythrocytes. J Exp Med *182*, 15-20.

Tse, M.T., Chakrabarti, K., Gray, C., Chitnis, C.E., and Craig, A. (2004). Divergent binding sites on intercellular adhesion molecule-1 (ICAM-1) for variant *Plasmodium falciparum* isolates. Mol Micro *51*, 1039-1049.

Waterkeyn, J.F., Wickham, M.E., Davern, K., Cooke, B.M., Reeder, J.C., Culvenor, J.G., Waller, R.F., and Cowman, A.F. (2000). Targeted mutagenesis of *Plasmodium falciparum* erythrocyte membrane protein 3 (PfEMP3) disrupts cytoadherence of malaria-infected red blood cells. EMBO J *19*, 2813-2823.

Southern Blots Maier *et al.* S1-1

PFA0110w

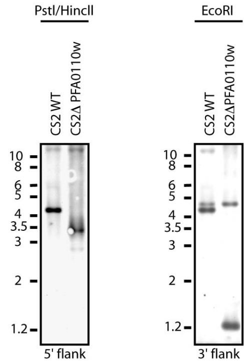

Expected sizes:		5' flank	3' flank
	WT	4.4 kb	4.3 kb
	ko	3.5 kb	1.2 kb
	plasmid	2.6 kb	9.2 kb

PFA0620c

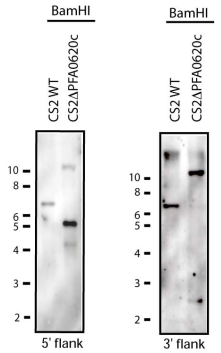

Expected sizes:		5' flank	3' flank
	WT	6.6 kb	6.6 kb
	ko	4.5 kb	2.6 kb
	plasmid	9.2 kb	9.2 kb

PFA0630c

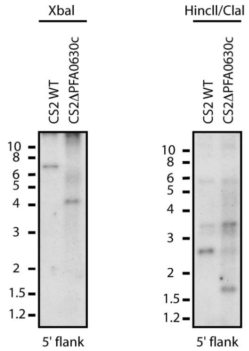

Expected sizes:		XbaI	HincII/ClaI
	WT	7.4 kb	2.7 kb
	ko	4.5 kb	1.7 kb
	plasmid	8.2 kb	9.1 kb

PFB0085c

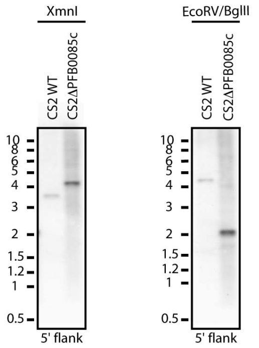

Expected sizes:		XmnI	EcoRV/BglII
	WT	3.7 kb	4.7 kb
	ko	4.5 kb	2.2 kb
	plasmid	8.1 kb	1.9 kb

PFB0090c

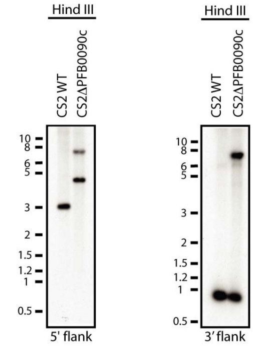

Expected sizes:		5' flank	3' flank
	WT	3.1 kb	0.9 kb
	ko	4.6 kb	1.4 kb
	plasmid	8.9 kb	8.9 kb

PFB0106c

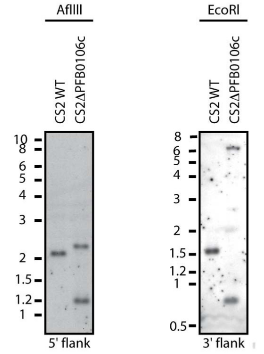

Expected sizes:		5' flank	3' flank
	WT	2.1 kb	1.6 kb
	ko	1.2 kb	0.8 kb
	plasmid	2.3 kb	6.1 kb

Southern Blots Maier *et al.* S1-2

PFB0915c

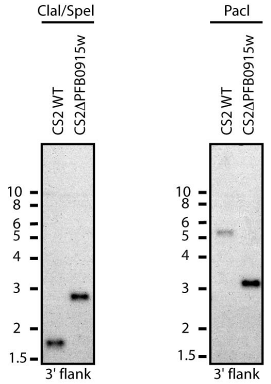

Expected sizes:		ClaI/SpeI	PacI
	WT	1.8 kb	5.3 kb
	ko	2.8 kb	3.2 kb
	plasmid	9.0 kb	9.0 kb

PFB0920w

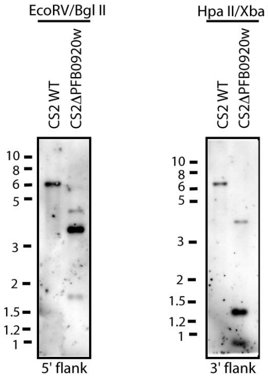

Expected sizes:		EcoRV/Bgl II	Hpa II/Xba I
	WT	6.2 kb	6.9 kb
	ko	3.6 kb	1.4 kb
	plasmid	1.7 kb	0.9 kb

PFB0925w

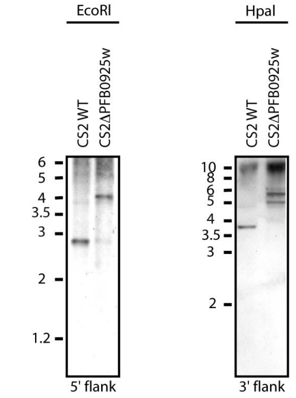

Expected sizes:		EcoRI	HpaI
	WT	2.8 kb	4.2 kb
	ko	4.3 kb	6.1 kb
	plasmid	8.6 kb	9.0 kb

PFD0090c

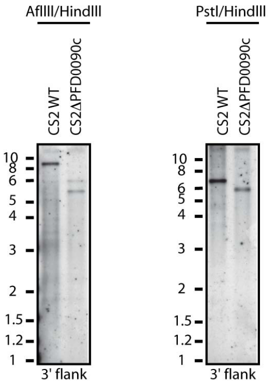

Expected sizes:		AflIII/HindIII	PstI/HindIII
	WT	9.5 kb	7.0 kb
	ko	5.8 kb	5.8 kb
	plasmid	2.0 kb	5.0 kb

PFD0225w

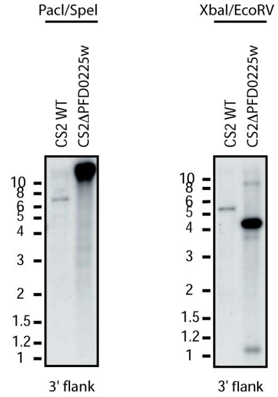

Expected sizes:		PacI/SpeI	XbaI/EcoRV
	WT	6.2 kb	5.8 kb
	ko	3.4 kb	1.8 kb
	plasmid	- kb	3.7 kb

PFD0320c

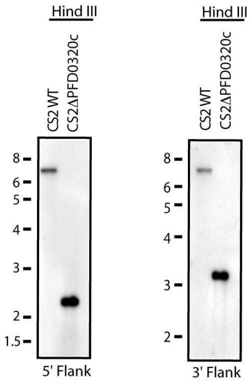

Expected sizes:		Hind III	Hind III
	WT	7.0 kb	7.0 kb
	ko	2.3 kb	3.3 kb
	plasmid	7.6 kb	7.6 kb

Southern Blots Maier *et al.* S1-3

PFD0495c

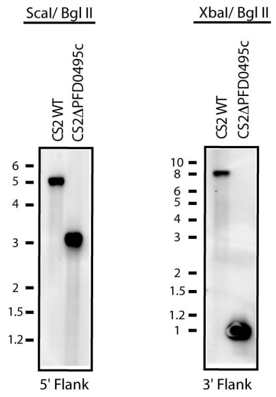

Expected sizes:		5' Flank	3' Flank
	WT	5.2 kb	8.0 kb
	ko	3.3 kb	0.9 kb
	plasmid	5.4 kb	7.0 kb

PFD1140w

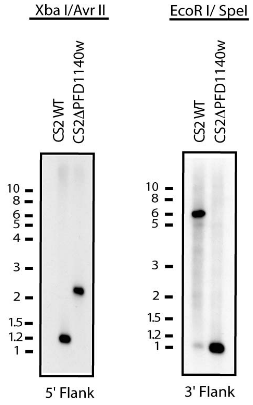

ected sizes:		5' Flank	3' Flank
	WT	1.2 kb	6.4 kb
	ko	2.2 kb	1.1 kb
	plasmid	7.8 kb	9.3 kb

PFD1160w

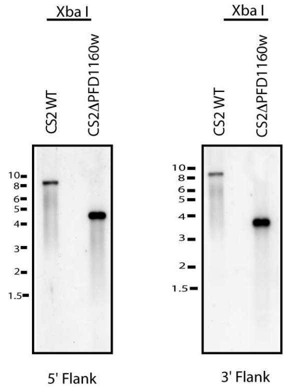

Expected sizes:		5' Flank	3' Flank
	WT	8.6 kb	8.6 kb
	ko	4.8 kb	3.6 kb
	plasmid	8.5 kb	8.5 kb

PFD1170c

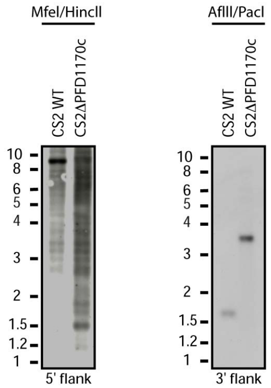

Expected sizes:		5' flank	3' flank
	WT	4.1 kb	1.7 kb
	ko	1.3 kb	3.5 kb
	plasmid	3.1 kb	9.2 kb

PFE0050w

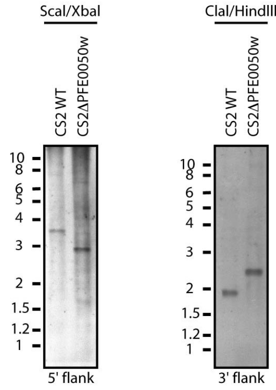

Expected sizes:		5' flank	3' flank
	WT	3.5 kb	2.0 kb
	ko	3.0 kb	2.5 kb
	plasmid	3.8 kb	5.0 kb

PFE0055c

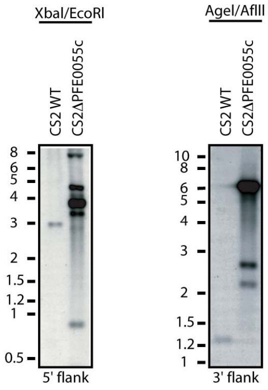

Expected sizes:		5' flank	3' flank
	WT	3.0 kb	1.3 kb
	ko	3.5 kb	2.8 kb
	plasmid	4.0 kb	6.4 kb

Southern Blots Maier *et al.* S1-4

PFE0060w

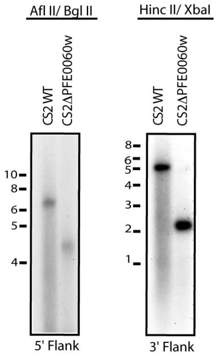

Expected sizes:		Afl II/ Bgl II	Hinc II/ XbaI
	WT	6.8 kb	5.3 kb
	ko	4.5 kb	2.3 kb
	plasmid	9.2 kb	7.1 kb

PFE0070w

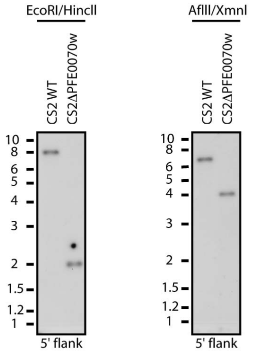

Expected sizes:		EcoRI/HincII	AflII/XmnI
	WT	8.0 kb	6.7 kb
	ko	2.1 kb	4.4 kb
	plasmid	7.0 kb	8.3 kb

MAL7P1.91

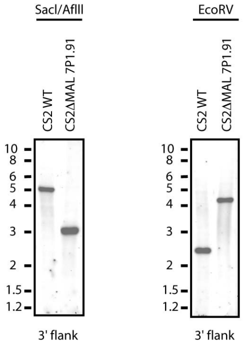

Expected sizes:		SacI/AflII	EcoRV
	WT	5.3 kb	2.5 kb
	ko	3.1 kb	4.4 kb
	plasmid	5.9 kb	5.8 kb

PF07_0107

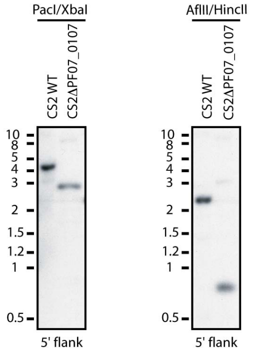

Expected sizes:		PacI/XbaI	AflII/HincII
	WT	4.1 kb	2.4k b
	ko	2.9 kb	0.8 kb
	plasmid	7.9 kb	8.8 kb

MAL7P1.153

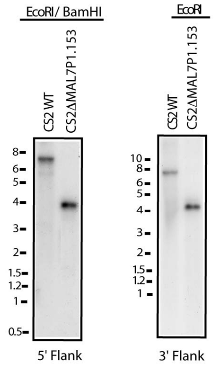

Expected sizes:		EcoRI/ BamHI	EcoRI
	WT	7.5 kb	7.5 kb
	ko	3.7 kb	4.5 kb
	plasmid	8.3 kb	8.3 kb

MAL7P1.170

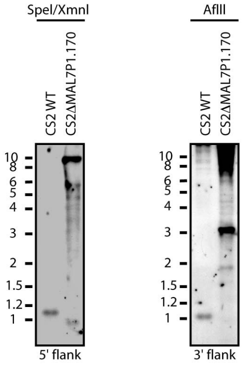

Expected sizes:		SpeI/XmnI	AflII
	WT	1.1 kb	1.0 kb
	ko	6.2 kb	3.1 kb
	plasmid	4.3 kb	9.1 kb

Southern Blots Maier *et al.* S1-5

MAL7P1.171

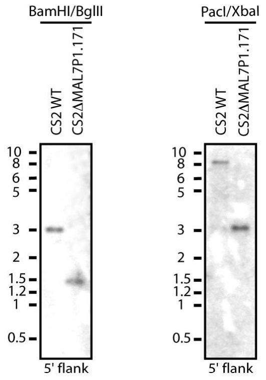

Expected sizes:		BamHI/BglII	PacI/XbaI
	WT	3.1 kb	8.8 kb
	ko	1.4 kb	3.1 kb
	plasmid	8.1 kb	8.3 kb

MAL7P1.172

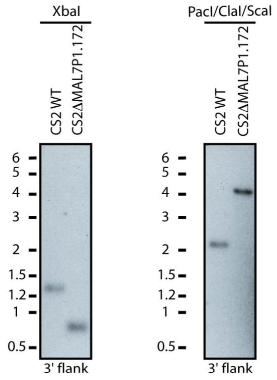

Expected sizes:		XbaI	PacI/ClaI/ScaI
	WT	1.3 kb	2.2 kb
	ko	1.6 kb	4.1 kb
	plasmid	8.2 kb	4.0 kb

MAL7P1.174

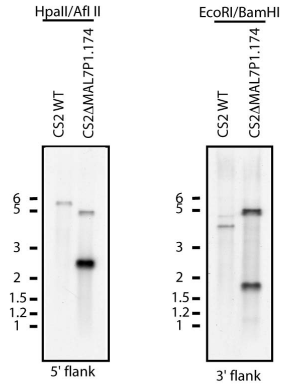

Expected sizes:		HpaII/Afl II	EcoRI/BamHI
	WT	5.8 kb	4.0 kb
	ko	4.9 kb	1.9 kb
	plasmid	2.5 kb	5.0 kb

MAL8P1.154

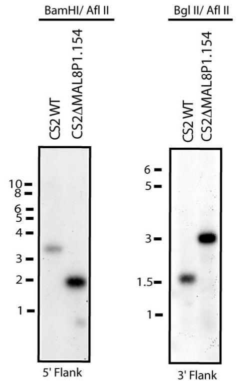

Expected sizes:		BamHI/ Afl II	Bgl II/ Afl II
	WT	3.2 kb	3.9 & 1.6 kb
	ko	2.1 kb	3.9 & 2.9 kb
	plasmid	7.1 kb	6.0 kb

PFI0260c

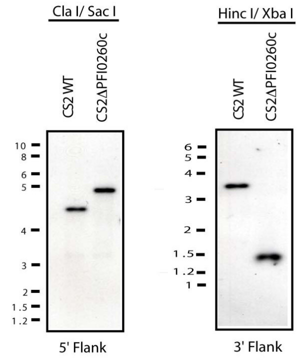

Expected sizes:		Cla I/ Sac I	Hinc I/ Xba I
	WT	3.9 kb	3.5 kb
	ko	4.8 kb	1.5 kb
	plasmid	5.6 kb	4.3 kb

PFI1755c

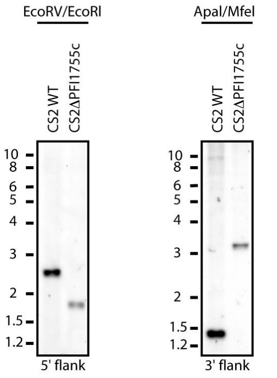

Expected sizes:		EcoRV/EcoRI	ApaI/MfeI
	WT	2.6 kb	1.5 kb
	ko	1.9 kb	3.3 kb
	plasmid	3.4 kb	6.3 kb

Southern Blots Maier *et al.* S1-6

PF10_0024

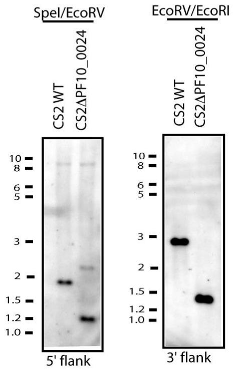

Expected sizes:		5' flank	3' flank
	WT	2.0 kb	3.2 kb
	ko	1.4 kb	2.0 kb
	plasmid	3.4 kb	5.5 kb

PF10_0025

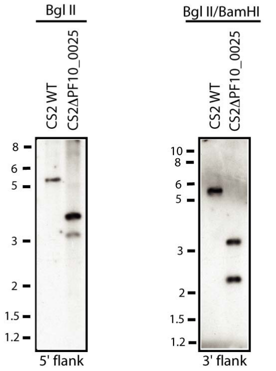

Expected sizes:		5' flank	3' flank
	WT	7.0 kb	7.0 kb
	ko	5.2 kb	2.5 kb
	plasmid	2.0 kb	6.1 kb

PF10_0159

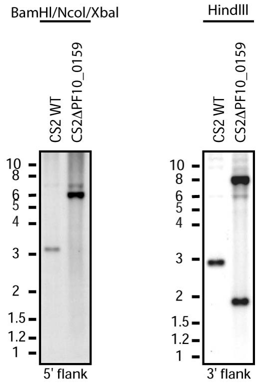

Expected sizes:		5' flank	3' flank
	WT	3.4 kb	2.8 kb
	ko	1.7 kb	1.9 kb
	plasmid	7.3 kb	7.5 kb

PF10_0378

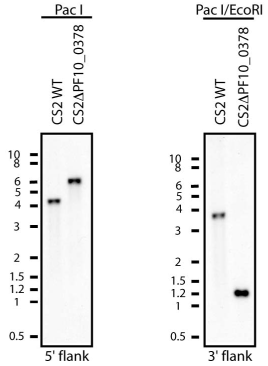

Expected sizes:		5' flank	3' flank
	WT	4.7 kb	3.8 kb
	ko	6.4 kb	1.2 kb
	plasmid	8.9 kb	4.1 kb

PF10_0381

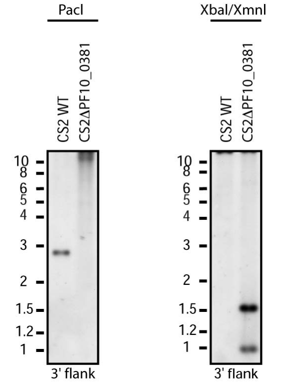

Expected sizes:		3' flank	3' flank
	WT	2.7 kb	3.8 kb
	ko	4.8 kb	1.0 kb
	plasmid	- kb	1.5 kb

PF11_0035

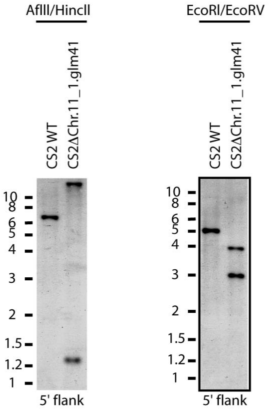

Expected sizes:		5' flank	5' flank
	WT	6.1 kb	4.7 kb
	ko	1.3 kb	3.0 kb
	plasmid	9.1 kb	2.0 kb

Southern Blots Maier *et al.* S1-7

PF11_0037

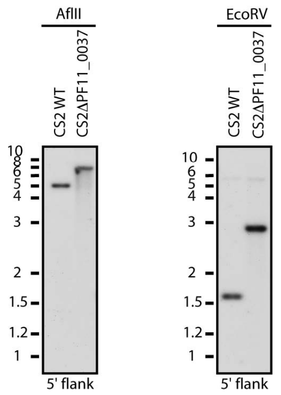

Expected sizes:		AflII	EcoRV
	WT	5.0 kb	1.6 kb
	ko	8.2 kb	2.9 kb
	plasmid	- kb	3.7 kb

PF11_0507

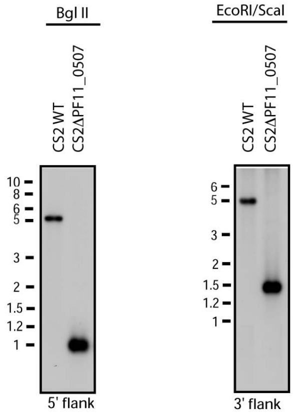

Expected sizes:		Bgl II	EcoRI/ScaI
	WT	3.6 kb	5.4 kb
	[illegible]	[illegible]	[illegible]
	plasmid	7.1 kb	1.7 kb

PF11_0512

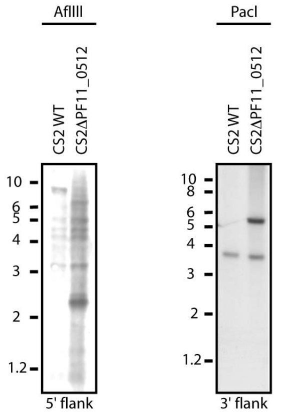

Expected sizes:		AflIII	PacI
	WT	8.0 kb	3.7 kb
	ko	2.3 kb	5.6 kb
	plasmid	2.5 kb	6.8 kb

PF11_0513

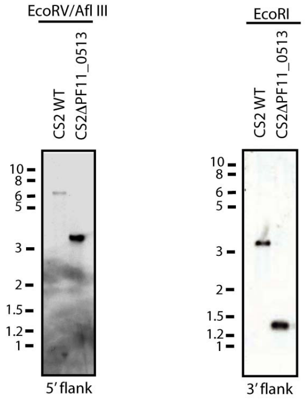

Expected sizes:		EcoRV/Afl III	EcoRI
	WT	6.0 kb	3.4 kb
	ko	4.2 kb	1.4 kb
	plasmid	2.0 kb	8.9 kb

PFL0050c

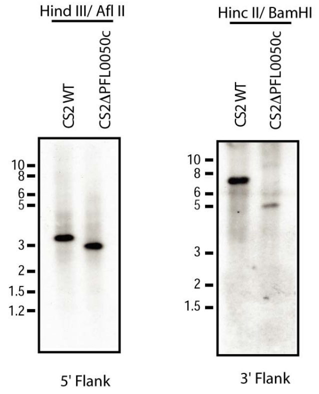

Expected sizes:		Hind III/ Afl II	Hinc II/ BamHI
	WT	3.4 kb	7.2 kb
	ko	3.0 kb	5.0 kb
	plasmid	6.1 kb	5.1 kb

PFL2550w

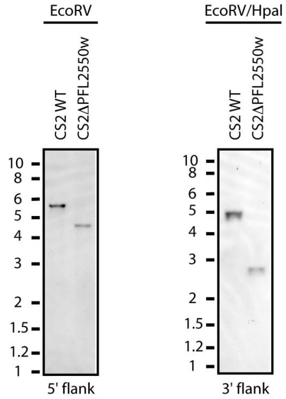

Expected sizes:		EcoRV	EcoRV/HpaI
	WT	5.8 kb	4.7 kb
	ko	4.7 kb	2.9 kb
	plasmid	3.4 kb	3.4 kb

Southern Blots Maier *et al.* S1-8

PF13_0073

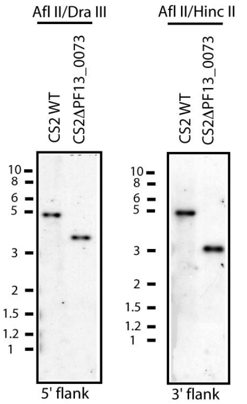

Expected sizes:		5' flank	3' flank
	WT	5.0 kb	5.1 kb
	ko	3.8 kb	3.1 kb
	plasmid	1.7 kb	6.0 kb

PF13_0076

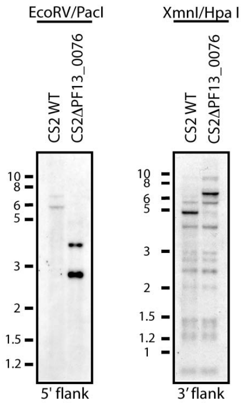

Expected sizes:		5' flank	3' flank
	WT	6.9 kb	5.3 kb
	ko	3.9 kb	7.2 kb
	plasmid	3.0 kb	8.9 kb

PF13_0275

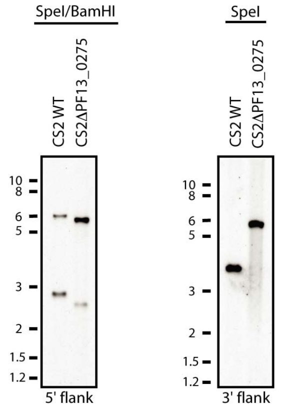

Expected sizes:		5' flank	3' flank
	WT	2.9 kb	3.9 kb
	ko	2.7 kb	6.1 kb
	plasmid	1.7 kb	8.7 kb

PF14_0018

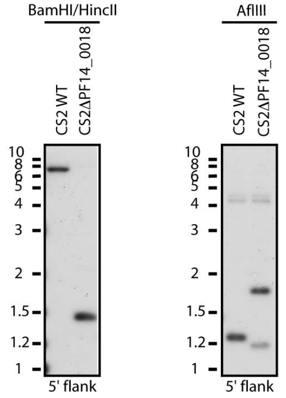

Expected sizes:		5' flank	5' flank
	WT	7.0 kb	1.2 kb
	ko	1.4 kb	1.8 kb
	plasmid	2.1 kb	2.8 kb

PF14_0152

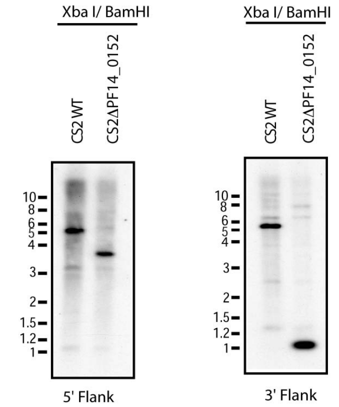

Expected sizes:		5' Flank	3' Flank
	WT	5.9 kb	5.9 kb
	ko	4.3 kb	1.2 kb
	plasmid	7.1 kb	7.3 kb

PF14_0236

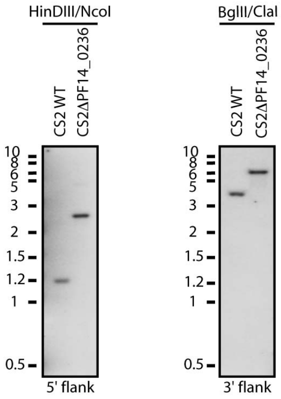

Expected sizes:		5' flank	3' flank
	WT	1.1 kb	3.3 kb
	ko	0.7 kb	5.3 kb
	plasmid	2.2 kb	6.7 kb

Southern Blots Maier *et al.* S1-9

PF14_0250

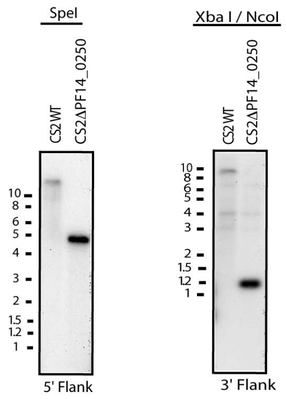

Expected sizes:		5' Flank	3' Flank
	WT	13.3 kb	10.9 kb
	ko	4.9 kb	1.2 kb
	plasmid	9.5 kb	8.7 kb

PF14_0271

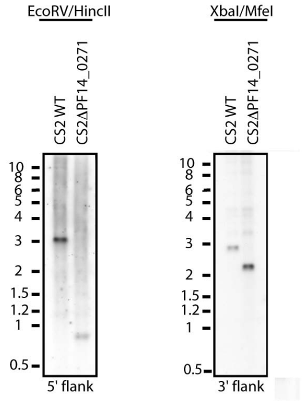

Expected sizes:		5' flank	3' flank
	WT	3.1 kb	2.9 kb
	ko	0.8 kb	2.3 kb
	plasmid	1.9 kb	4.0 kb

PF14_0404

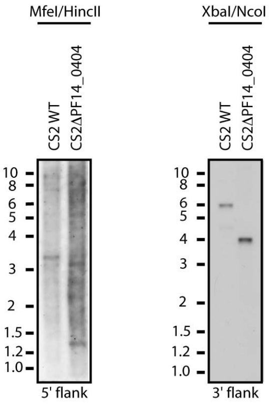

Expected sizes:		5' flank	3' flank
	WT	3.6 kb	6.2 kb
	ko	1.5 kb	4.0 kb
	plasmid	3.1 kb	8.6 kb

PF14_0588

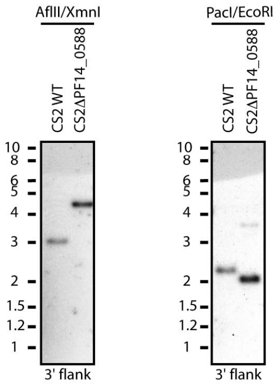

Expected sizes:		3' flank (AflII/XmnI)	3' flank (PacI/EcoRI)
	WT	3.1 kb	2.4 kb
	ko	4.8 kb	2.1 kb
	plasmid	9.5 kb	9.5 kb

PF14_0758

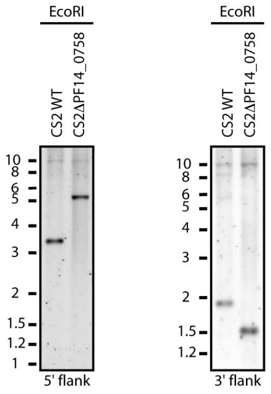

Expected sizes:		5' flank	3' flank
	WT	3.5 kb	2.3 kb
	ko	5.6 kb	1.8 kb
	plasmid	9.1 kb	9.1 kb

Maier *et al.*
Figure S2

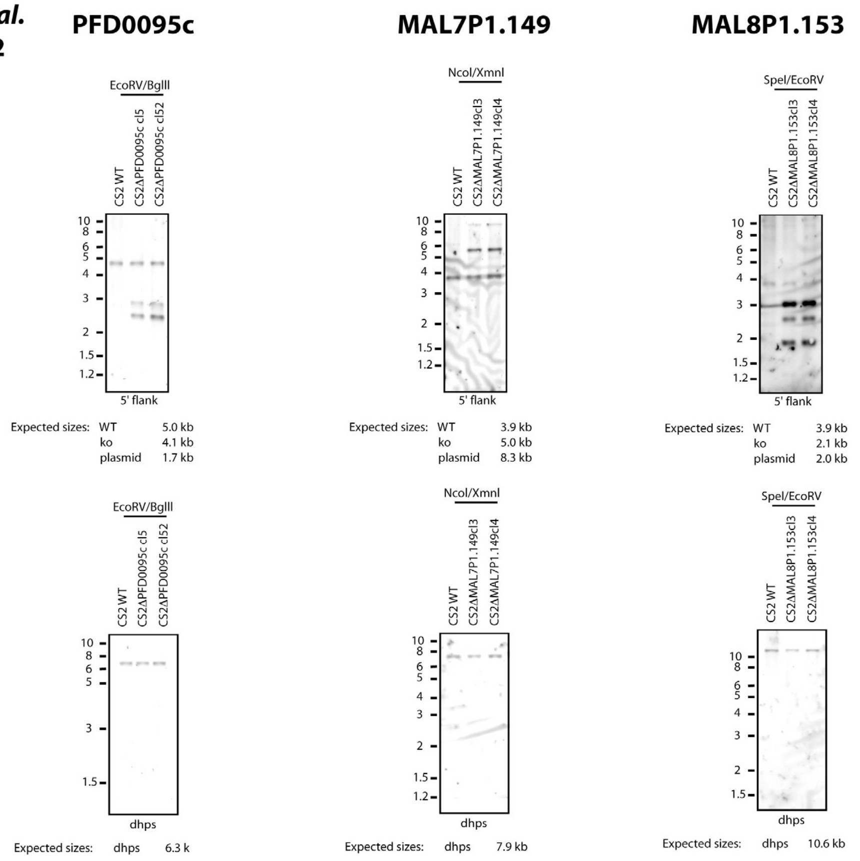

Maier *et al.*
Figure S3

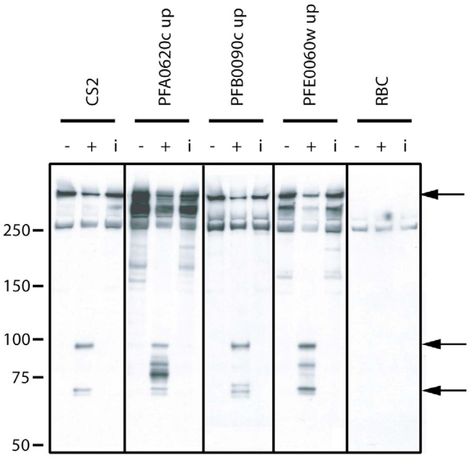

Maier *et al.*
Supplementary
Figure S4

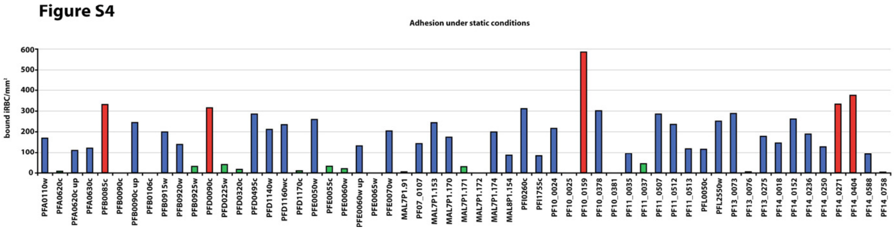

Maier *et al.*
Supplementary
Figure 5

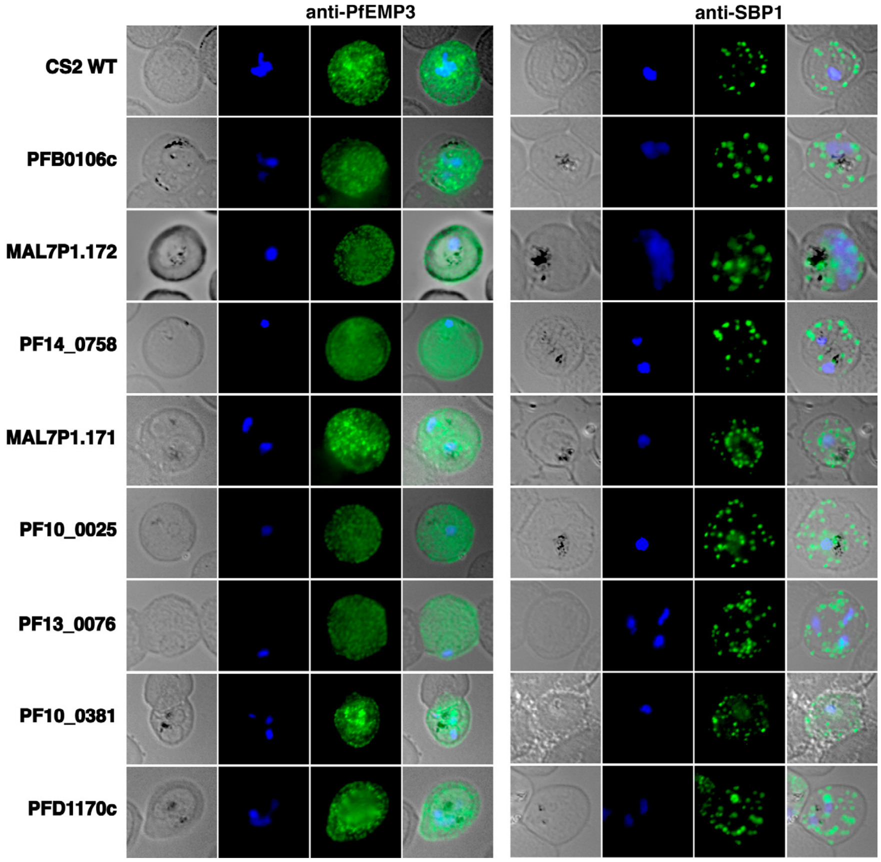

Maier *et al.*
Figure S6

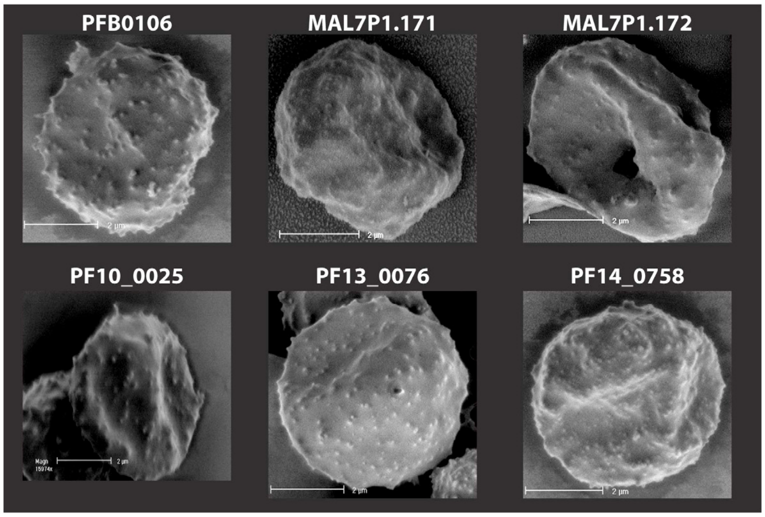

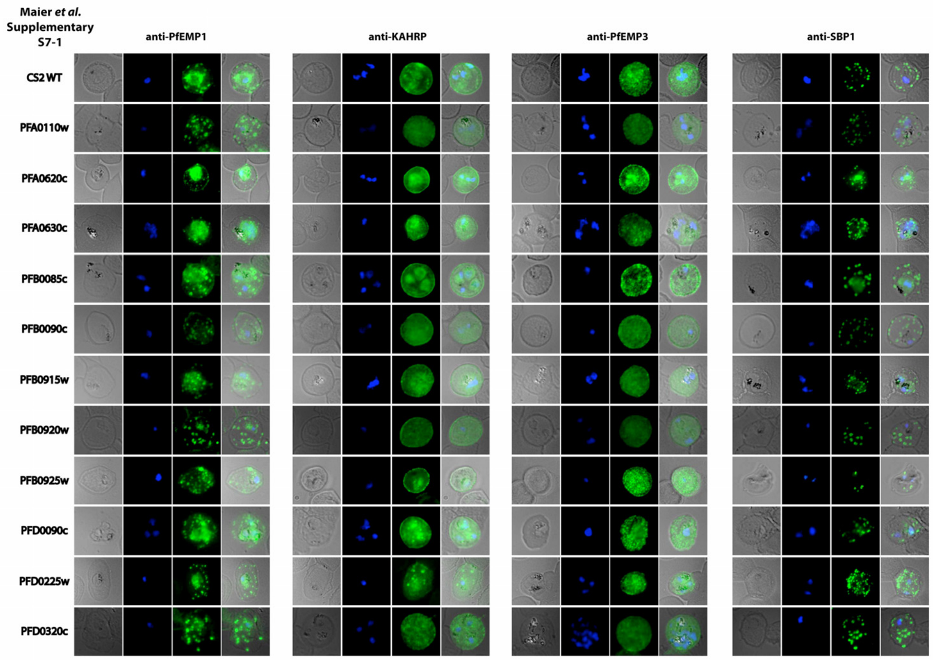
Maier et al.
Supplementary
S7-1
anti-PfEMP1
anti-KAHRP
anti-PfEMP3
anti-SBP1
CS2 WT
PFA0110w
PFA0620c
PFA0630c
PFB0085c
PFB0090c
PFB0915w
PFB0920w
PFB0925w
PFD0090c
PFD0225w
PFD0320c

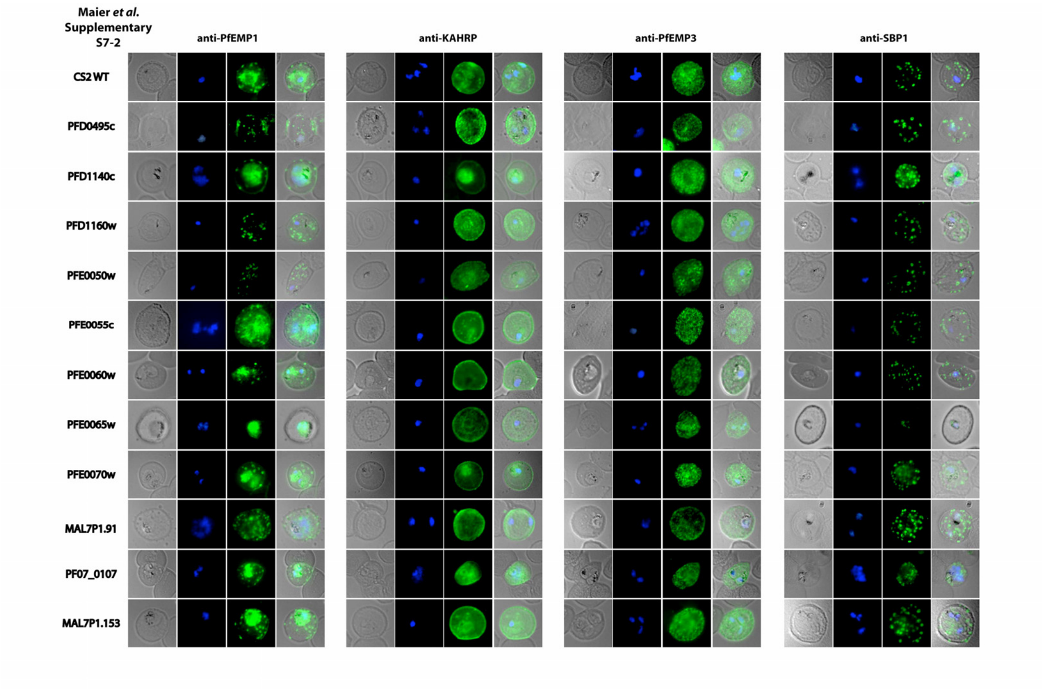
Maier et al.
Supplementary
S7-2
anti-PfEMP1
anti-KAHRP
anti-PfEMP3
anti-SBP1
CS2 WT
PFD0495c
PFD1140c
PFD1160w
PFE0050w
PFE0055c
PFE0060w
PFE0065w
PFE0070w
MAL7P1.91
PF07_0107
MAL7P1.153

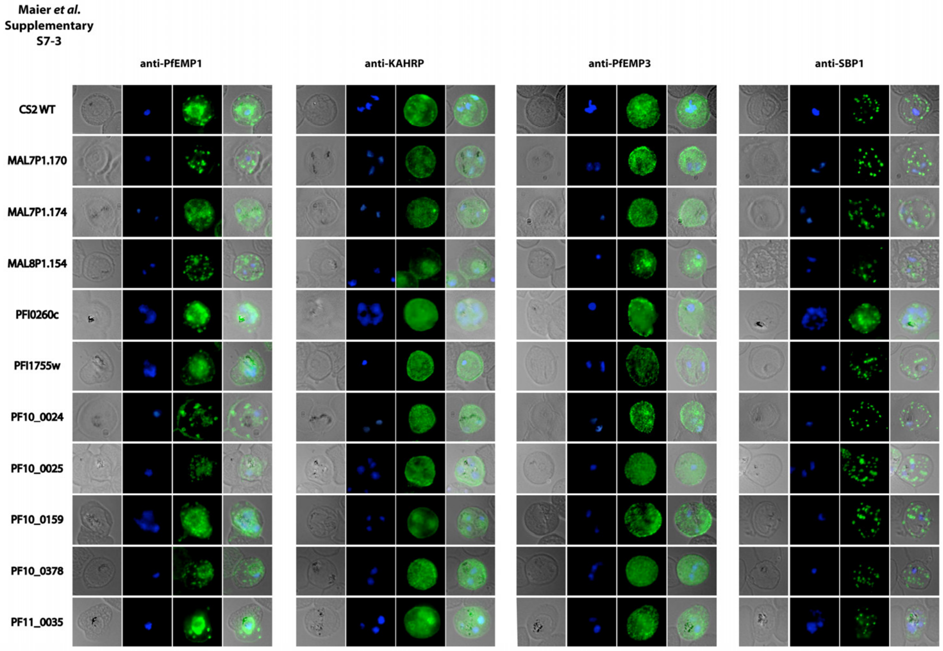
Maier et al.
Supplementary
S7-3
anti-PfEMP1
anti-KAHRP
anti-PfEMP3
anti-SBP1
CS2 WT
MAL7P1.170
MAL7P1.174
MAL8P1.154
PFI0260c
PFI1755w
PF10_0024
PF10_0025
PF10_0159
PF10_0378
PF11_0035

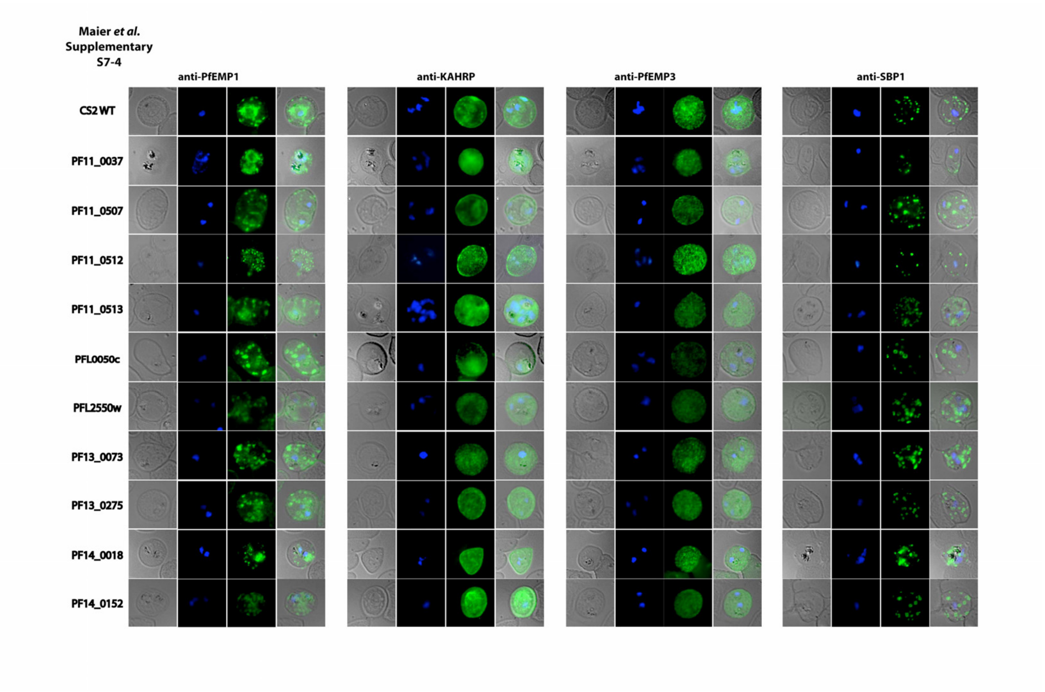

Maier *et al.*
Supplementary
S7-4
anti-PfEMP1
anti-KAHRP
anti-PfEMP3
anti-SBP1
CS2 WT
PF11_0037
PF11_0507
PF11_0512
PF11_0513
PFL0050c
PFL2550w
PF13_0073
PF13_0275
PF14_0018
PF14_0152

Maier *et al.*
Supplementary
S7-5

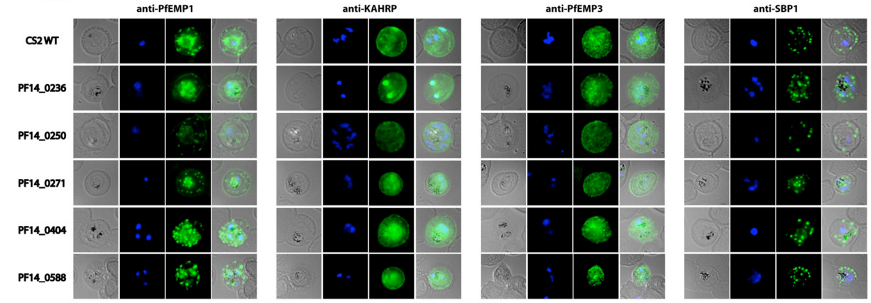

Supplement: Document S1. Supplemental Experimental Procedures, Nineteen Figures, Three Tables, and Supplemental References [file mmc1.pdf]
